# Exosome-transmitted long noncoding RNA SNHG1 promotes prostate cancer bone metastasis via YBX1/MMP16 axis

**DOI:** 10.1038/s41420-025-02855-5

**Published:** 2026-01-08

**Authors:** Taowei Yang, Junqi Luo, Zining Long, Jun Wu, Wenbin Chen, Xumin Zhou, Libin Zou, Shengren Cen, Chuanfan Zhong, Jianming Lu, Pengxiang Zheng, Anyang Wei, Daojun Lv, Xiangming Mao

**Affiliations:** 1 Department of Urology, The First Affiliated Hospital, Sun Yat-sen University, Guangzhou, China; 2 Department of Urology, Nanfang Hospital, Southern Medical University, Guangzhou, China; 3 Department of Urology, Zhujiang Hospital, Southern Medical University, Guangzhou, China; 4 Department of Urology, Shanghai Ninth People’s Hospital, Shanghai Jiao Tong University School of Medicine, Shanghai, China; 5 General Surgery Center, Department of Thyroid Surgery, Zhujiang Hospital, Southern Medical University, Guangzhou, China; 6 Department of Andrology, Guangzhou First People’s Hospital, Guangzhou Medical University, Guangzhou, Guangdong China; 7 Department of Urology, Fuqing City Hospital Affiliated to Fujian Medical University, Fuzhou, Fujian China; 8 Department of Urology, The Third Affiliated Hospital of Guangzhou Medical University, Guangzhou, China

**Keywords:** Bone metastases, Prostate cancer, Cancer models

## Abstract

Prostate cancer (PCa) patients with bone metastasis commonly exhibit osteoblastic-type and have an extremely poor prognosis. Exosomes derived from tumor cells possess biological significance and can mediate intercellular communication in the tumor microenvironment. Long noncoding RNA (lncRNA) small nucleolar RNA host gene 1 (SNHG1) is implicated in tumorigenesis and the development of PCa, but the precise roles of SNHG1 in the regulation of bone homeostasis remain elusive. Herein, we aimed to investigate the underlying mechanisms by which exosomes-encapsulated SNHG1 affects the bone metastasis of PCa. Our findings revealed that SNHG1 was overexpressed in PCa tissues, highly enriched in PCa cell-derived exosomes, and positively correlated with bone metastasis. Besides, SNHG1 shuttled by PCa-derived exosomes could be transferred into osteoblast cells, where SNHG1 exerted inductive properties in osteogenic differentiation. Gain- and loss-of-functional experiments demonstrated that exosomal SNHG1 facilitated the activity of alkaline phosphatase and mineralization of extracellular matrix. Moreover, in vivo experimentation showed that knockdown of exosomal SNHG1 suppressed bone metastasis of PCa cells. Mechanistic investigations revealed that exosomal SNHG1, transmitted to osteoblast cells, physically binds to YBX1 and leads to the shift of YBX1 into the nucleus, then enhances MMP16 transcription and increases the amount of protein translation, ultimately resulting in PCa bone metastasis. In conclusion, our data highlight that PCa-derived exosomes-loaded SNHG1 mediated osteogenesis through the SNHG1/YBX1/MMP16 axis. SNHG1 may serve as a potential diagnostic marker and therapeutic target for bone metastasis in PCa.

## Introduction

According to the 2020 World Health Organization Epidemiology statistics, prostate cancer (PCa) is the second most prevalent malignancy in men worldwide [1]. Currently, at the fifth leading cause of male tumor death, PCa mortality has declined in most high-income countries since the mid-1990s, due to advances in PCa screening and early diagnosis [1, 2]. Even so, the most prominent contributor to PCa-related deaths is tumor metastasis. The 5-year survival rate for localized or regional PCa can approach 100%, but for those with distant metastasis, the 5-year survival rate is only 30% [3]. Among them, bone metastasis (BM) is the most common of the distant metastasis from PCa. Treatment options for PCa bone metastasis are limited and often provide only symptomatic relief [2]. Hence, it is imperative to better investigate the pathways and underlying molecular mechanisms of PCa bone metastasis.

Stephen Paget proposed a “seed and soil” theory to explain the distant metastasis of tumors to specific target organs, arguing that tumor metastasis is not random [4]. Cancer cells are like “seeds” and the bone microenvironment is like “soil”, and the mutual molecular communication between cancer cells and bone microenvironment creates the affinity of PCa for bone tissue [5]. While bone metastasis from other cancers, such as breast and lung cancer, often results in osteolytic lesions, most of the PCa bone metastasis uniquely induces osteogenic changes [6]. Recently, there has been an increasing interest in the role of exosomes in intercellular communication in cancer metastasis. Exosomes are extracellular vesicles with a diameter of 30–150 nm and can contain nucleic acids, lipids, and proteins, which can be safely and stably transported to distant tissues [7]. Previous studies have reported that tumor-derived exosomes contribute to the initial communication between the primary tumor and the metastatic site [8]. More surprisingly, Hoshino et al. found that exosomes of specific tumor origin are preferentially taken up by their common metastatic organs and communicate with pre-metastatic niches [9]. In a sense, the exosomes may be the real seeds in the “seed and soil” theory. This suggests that PCa exosomes may have a bone tissue propensity and specificity for bone tissue regulation and may be a key mediator in the modification of the microenvironment prior to PCa bone metastasis.

The long noncoding RNA (lncRNA) small nucleolar RNA host gene 1 (SNHG1), located in the 11q12.3 region of the chromosome (GenBank accession ID: 23642), was aberrantly upregulated in several cancers and promotes malignant progression in lung [10], osteosarcoma [11, 12], and liver [13]. SNHG1 is highly expressed in the plasma of lung cancer [14] and liver cancer [15] patients, with the potential to function as a diagnostic marker. More importantly, SNHG1 is implicated in the malignant progression and development of PCa. Our previous study unveiled that SNHG1 participates in the epithelial–mesenchymal transition (EMT) process of PCa via SNHG1-hnRNPL-CDH1 axis [16]. Meanwhile, exosomal SNHG1 has also been highlighted to act as a ceRNA, where it has the potency to competitively bind to tumor-suppressive microRNAs (miRNAs) [17]. However, whether SNHG1 plays a role in the bone metastasis of PCa remains unexplored.

In this study, we found that the PCa-derived exosomal SNHG1 promoted bone metastasis by binding the YBX1 protein and inducing its nuclear localization to promote MMP16 transcription in osteoblasts. Highly expressed MMP16 activated osteoblasts and PCa colonization. Based on these findings, it can be concluded that SNHG1/YBX1/MMP16 axis could serve as a potential therapeutic target for PCa bone metastasis.

## Results

### Identification of SNHG1 as a bone metastasis-relevant lncRNA in PCa

**Fig. 1:**
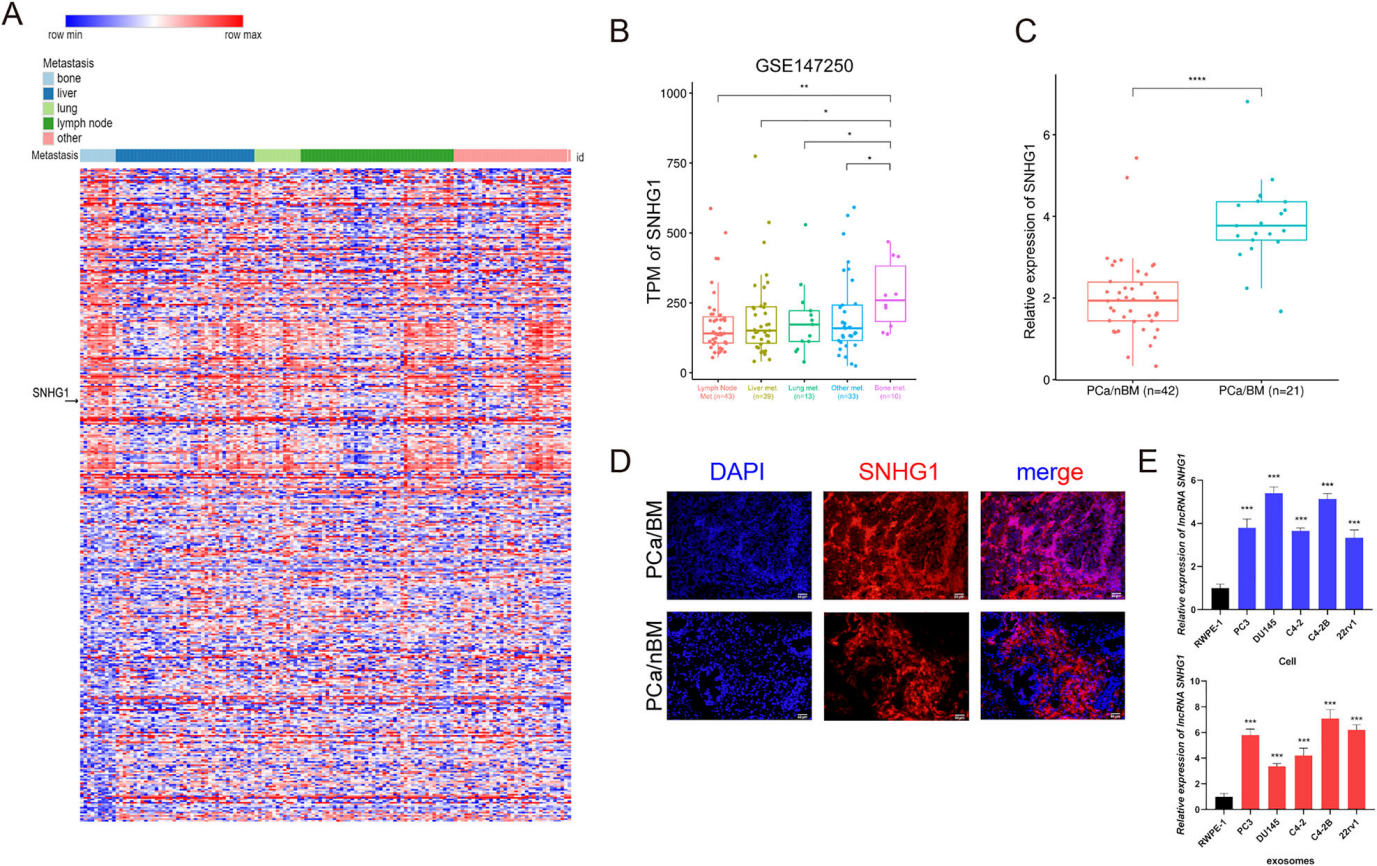
Identification of SNHG1 as a bone metastasis-relevant lncRNA in PCa. **A** Heatmaps showing the top differentially expressed lncRNAs in PCa metastasis tissues (bone *n* = 10, liver *n* = 39, lung *n* = 13, lymph node *n* = 43, other *n* = 33) of the GSE147250 dataset. The red shades represent high expression, and the blue shades represent low expression. **B** Box and whisker plot showing SNHG1 upregulated in bone metastatic prostate cancer tissues (GSE147250). The levels of SNHG1 were normalized to TPM. **C** SNHG1 expression in PCa patients (w/o Bone metastasis) plasma exosomes from Southern Medical University of Zhujiang Hospital. **D** The distribution of SNHG1 in PCa tissue determined by Fluorescence in situ hybridization (FISH). The scale bar indicates 50 μm. **E** SNHG1 expression in RWPE-1 and PCa cells or exosomes. SNHG1 expression measured by RT-qPCR, normalized to GAPDH (**C**, **E**). Error bars represent means ± SD; *p < 0.05, **p < 0.01, ***p < 0.001.

SNHG1 is highly expressed as an oncogenic lncRNA in several cancers (Fig. S1A). To determine the clinical significance of SNHG1 in PCa and bone metastasis, we analyzed the lncRNA expression profiles using the Cancer Genome Atlas (TCGA) dataset and Gene Expression Omnibus (GEO) dataset (GSE147250). TCGA database showed that SNHG1 was overexpressed in PCa compared to normal tissues (Fig. S1B), and PCa patients with high SNHG1 expression had poor overall and disease-free survival (Fig. S1C, D). To further investigate whether SNHG1 is associated with PCa bone metastasis, we analyzed the differentially expressed genes (DEGs) of GSE147250 and plotted the clustering heat map of the top 5% DEGs. The result showed that SNHG1 was in the top 5% upregulated genes in bone metastasis PCa (Fig. 1A). The Box plot demonstrated that expression of SNHG1 was significantly higher in bone metastasis PCa samples than in other PCa metastasis (Fig. 1B). Exosomes have been shown to have important functions in tumor progression, especially in distant tumor metastasis. This prompted us to evaluate whether exosomes from PCa can influence the bone metastatic process. We then evaluated SNHG1 expression levels in patients' plasma exosomes. RT-qPCR assays showed that SNHG1 is significantly upregulated in plasma exosomes of patients with PCa bone metastasis compared to primary PCa patients (Fig. 1C). In addition, we examined SNHG1 expression in PCa samples by RNA-FISH and showed that SNHG1 had significantly overexpression in PCa bone metastasis samples (Fig. 1D). Furthermore, consistent with the cell expression trend, the PCa cell lines also secreted significantly higher expression of exosomal SNHG1 compared to RWPE-1 (Normal prostate epithelial cell lineage) cells (Fig. 1E). The above plasma exosomes and C4-2B cell exosomes to detect SNHG1 were confirmed by sanger sequencing (Fig. S1E). Taken together, SNHG1 is highly expressed in PCa bone metastasis relative to other organ metastasis, and plasma exosomes from patients with PCa bone metastasis also overexpress SNHG1. SNHG1 may be involved in the PCa bone metastasis process through the exosomal pathway.

### PCa-derived exosomes promote osteoblast viability in vitro

**Fig. 2:**
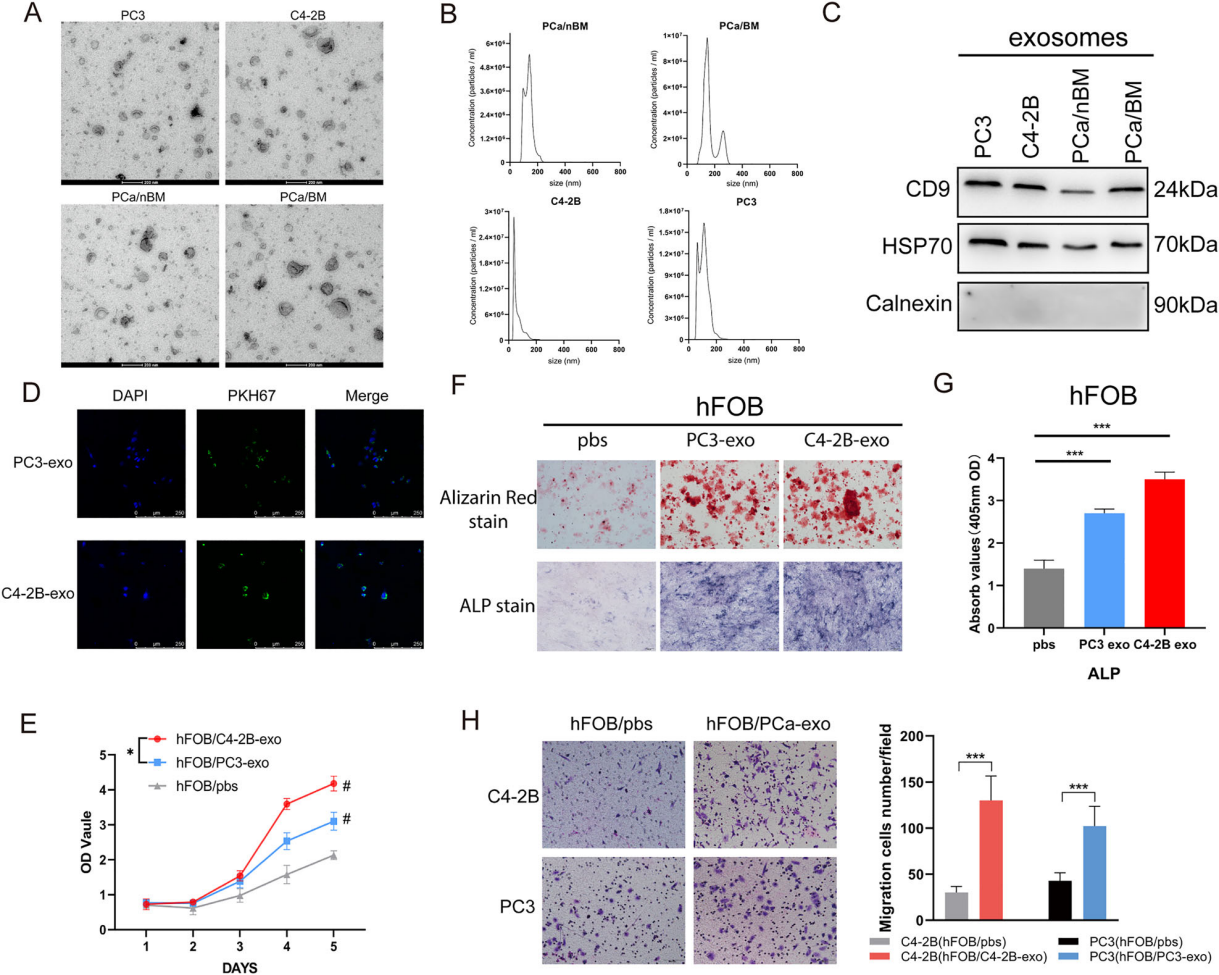
PCa-derived exosomes promote osteoblast viability in vitro. **A** Electron micrographs of exosomes isolated from the indicated samples. Exosomes of ~30–150 nm in diameter are shown. The scale bar indicates 200 nm. **B** Size distribution was determined using Nanoparticle Tracking Analysis (NTA). **C** Western blotting analysis of CD9, HSP70 and Calnexin in exosomes from PC3/C4-2B cell medium and PCa patient’s non-bone metastasis/bone metastasis (nBM/BM) plasma. **D** PKH67-labeled PCa cell-derived-exosomes entered hFOB observed by fluorescence microscope. PKH67-labeled exosomes were green, DAPI-stained nucleus was blue. The scale bar indicates 250 μm. **E** CCK8 assay detected the proliferation of hFOB treated with PBS, PC3-exosome or C4-2B-exosome for 5 days. # indicates statistical comparison with hFOB/PBS control group. **F** Alizarin red stain or Alkaline phosphatase (ALP) stain of hFOB treated with PBS or PCa-exo. **G** Detection of alkaline phosphatase (ALP) activity in hFOB treated with PCa exosomes. **H** Transwell assays were used to evaluate the ability of hFOB, after uptake of PCa cell exosomes, to induce migration of PC3 or C4-2B. Error bars represent means ± SD; ^#^*p* < 0.05, *p < 0.05, ***p < 0.001.

To evaluate whether PCa exosomes impact bone metastasis, exosomes were first isolated from PCa cell lines and patients' plasma, and the exosomes were characterized. Observed under transmission electron microscopy (TEM), the exosomes showed typical bilayer membrane particles with diameters in the range of 30–150 nm (Fig. 2A). NTA analysis revealed that exosomes were harvested more than 1 × 10^7^/ml and exosomes size were ~30–200 nm (Fig. 2B). Meanwhile, the expression of exosome positive markers (including CD9 and HSP70) and negative markers (Calnexin) was also confirmed by Western blot analysis (Fig. 2C). Taken together, these data confirm that this population is predominantly exosomes, although some non-exosomal microvesicles are present [18].

Previous study confirmed that PCa communicates with osteoblasts via exosomal RNA and enhances osteoblasts (hFOB) proliferation and activity as a potential new pathway to mediate PCa bone metastasis [19]. To explore the effect of PCa-derived exosomes on hFOB (hFOB1.19 cell line), PKH67-labeled PC3, C4-2B cell-derived exosomes were co-cultured with hFOB for 18 h. Subsequently, PCa-derived exosomes were found to enter the hFOB and to be distributed in the cytoplasm (Fig. 2D). Then, hFOB were treated with exosomes isolated from PC3 and C4-2B to observe the activity of hFOB. CCK8 assay revealed enhanced proliferation of hFOB that had ingested PCa exosomes (Fig. 2E). After 14 days of adding exosomes to the culture media, alizarin red staining test revealed increased bone nodules after ingestion of PCa exosomes (Fig. 2F). Alkaline phosphatase (ALP) staining and activity of hFOB treated with PCa-derived exosomes were found to be significantly higher than those in untreated hFOB (Fig. 2F, G). In further, enhanced activity of hFOB cells due to uptake of PCa exosomes, in turn, promoted the migratory ability of PCa cells (Fig. S2A and Fig. 2H). Moreover, exosomes derived from C4-2B stimulated a more pronounced increase in hFOB activity than exosomes derived from PC3 (Fig. 2E–H). These findings suggest that exosomes can enhance hFOB activity, with more pronounced effects of exosomes from osteogenic PCa cells (C4-2B) relative to osteolytic PCa cells (PC3).

### PCa-derived exosomes enhanced SNHG1 expression in hFOB

Since SNHG1 is highly expressed in PCa bone metastasis than all other PCa metastasis (Fig. 1B). SNHG1 was highly expressed in plasma exosomes of PCa bone metastasis (Fig. 1C) as well as in various PCa cellular exosomes (Fig. 1E). Also, PCa-derived exosomes can interact with osteoblasts. Therefore, the presence of SNHG1 in PCa-exosomes and the effect of SNHG1 on hFOB were further investigated in this study.

**Fig. 3:**
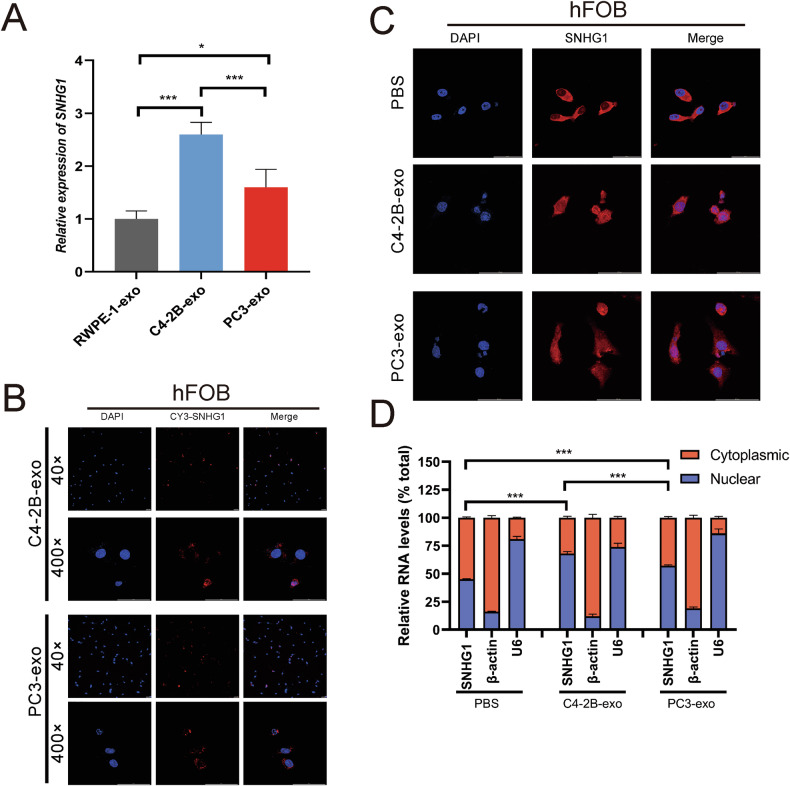
PCa-derived exosomes enhanced SNHG1 expression in hFOB. **A** SNHG1 expression in exosomes derived from RWPE-1 cells, C4-2B and PC3 cells cultured medium was measured by RT-qPCR, normalized to exogenous reference λpolyA. **B** The uptake of cy3-SNHG1 PCa-exosomes by hFOB was observed under the fluorescence microscope. The scale bar indicates 50 μm. **C** The localization of SNHG1 in hFOB co-cultured with PCa-exosomes was determined by FISH assay. The scale bar indicates 50 μm. **D** Expression levels of SNHG1 in subcellular fractions of hFOB cells after co-culture with PCa-exosomes were detected by RT-qPCR. U6 and β-actin were used as nuclear and cytoplasmic markers, respectively. U6 or ACTB was used as a loading control in RT-qPCR. Error bars represent means ± SD, *p < 0.05, ***p < 0.001.

First, hFOB was co-cultured with RWPE-1 and PCa exosomes to observe SNHG1 performance in hFOB. Compared with RWPE-1 exosomes, both C4-2B exosomes and PC3 exosomes elevated SNHG1 expression in hFOB, especially under C4-2B exosomes treatment (Fig. 3A). Next, to confirm that SNHG1 can be transported from PCa cells to hFOB via exosomes, we transfected PCa cells with cy3-labeled SNHG1 and harvested exosomes after 72 hours. The exosomes of cy3-SNHG1 were co-cultured with hFOB for 24 h. Fluorescence microscopy showed that cy3-SNHG1 was taken up by hFOB. And uptake of C4-2B-derived exosomes by hFOB, cy3-SNHG1 was more distributed in the nucleus, compared to PC3-derived exosomes (Fig. 3B). In addition, the localization of SNHG1 was examined by RNA-FISH, and it was also found that the PCa-derived SNHG1 taken up by hFOB was more localized in the nucleus (Fig. 3C). Meanwhile, nuclear-cytoplasmic segregation assays showed a higher distribution of SNHG1 in the nucleus after hFOB uptake of PCa-derived exosomes, especially those of C4-2B-derived (Fig. 3D). In conclusion, these findings suggest that SNHG1 in PCa can be transferred to hFOB via exosomes and translocated to the nucleus.

### SNHG1 shuttled by PCa-exosomes promoted osteoblast viability

To further verify whether the upregulation of SNHG1 in hFOB promotes hFOB activity, we treated hFOBs with exogenous overexpressed SNHG1 (Fig. S2B). It was found that overexpressed SNHG1 enhanced the proliferation of hFOB (Fig. S2C) and also significantly promoted the ALP expression of hFOB (Fig. S2D, E). In addition, alizarin red staining also showed that hFOB mineralization was significantly increased after SNHG1 overexpression. (Fig. S2E).

**Fig. 4:**
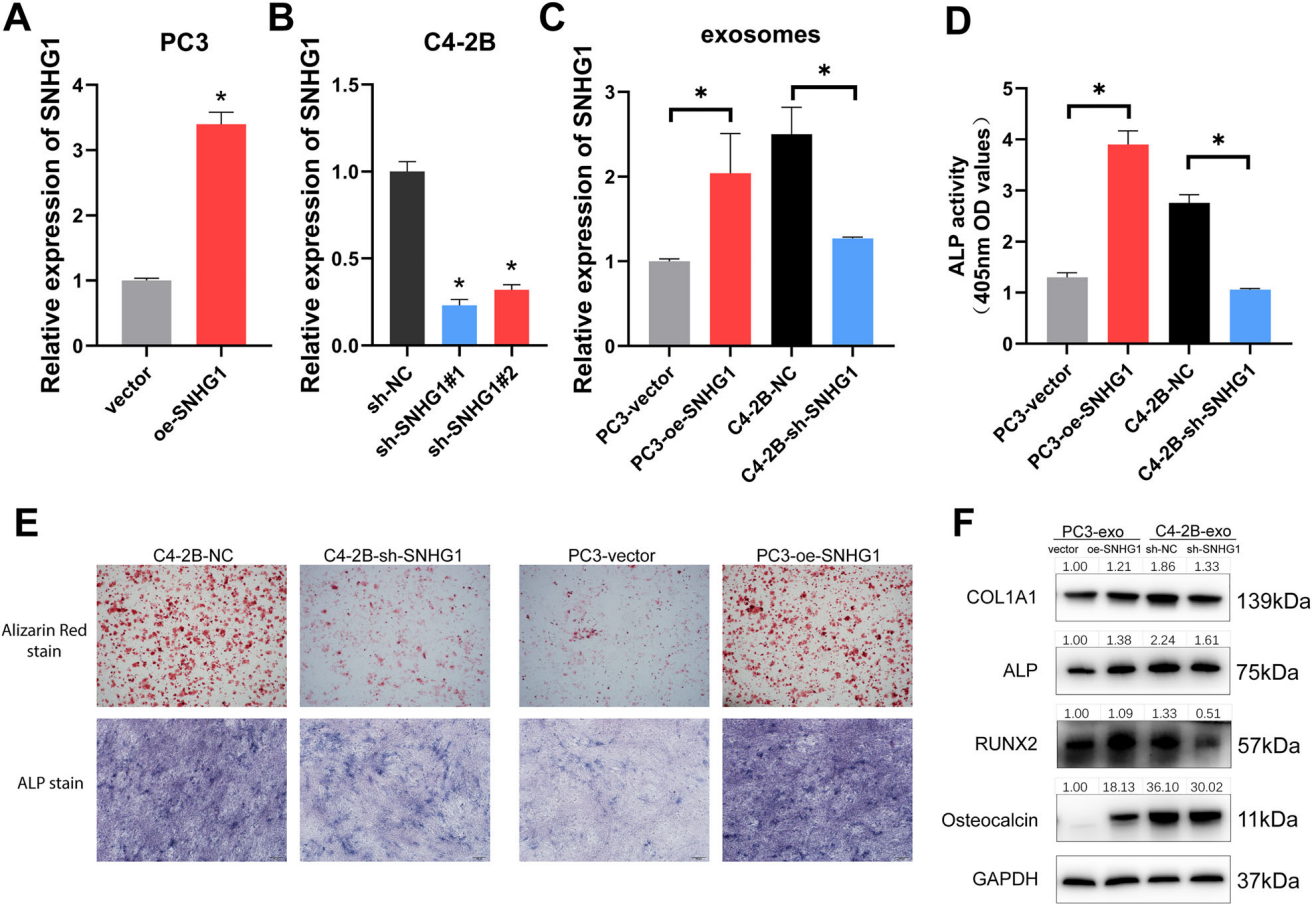
SNHG1 shuttled by PCa-exosomes promoted osteoblast viability. **A**, **B** SNHG1 expression was determined by RT-qPCR in PC3 or C4-2B with SNHG1 overexpression or knockdown, normalized to GAPDH. **C** SNHG1 expression in exosomes derived from PC3 or C4-2B cells with overexpression or knockdown of SNHG1 measured by RT-qPCR, normalized to exogenous reference λpolyA. **D** Detection of ALP activity in hFOB treated with PCa exosomes. **E** Alizarin red stain or Alkaline phosphatase stain of hFOB treated with PCa exosomes. **F** Western blotting analysis of COL1A1, ALP, RUNX2, Osteoclalcin of hFOB treated with PCa exosomes. Protein expression grayscale values were analyzed by imageJ, normalized to GAPDH. Error bars represent means ± SD, *p < 0.05.

Similarly, to verify that SNHG1 exhibited the same effect on hFOB in PCa-exosomes, SNHG1 was knocked down or overexpressed in C4-2B and PC3 cells, respectively (Fig. 4A, B). and extracted exosomes with high or low SNHG1 expression (Fig. 4C). The ALP staining, activity and mineralization were significantly stronger in hFOBs with higher expression of SNHG1 (PC3-SNHG1-exo and C4-2B-sh-NC-exo) than in hFOBs with lower expression of SNHG1 (PC3-NC-exo and C4-2B-sh-SNHG1-exo; Fig. 4D, E). In addition, protein levels of RUNX2, ALP, COL1A1 and Osteocalcin, which are osteogenic markers, were also highly expressed in PCa-exosomes with elevated SNHG1 (Fig. 4F). Collectively, these findings suggest that SNHG1 transferred by PCa-exosomes enhances hFOB osteogenesis.

### SNHG1 interacts with the transcription factor YBX1 and induces YBX1 into the nucleus

**Fig. 5:**
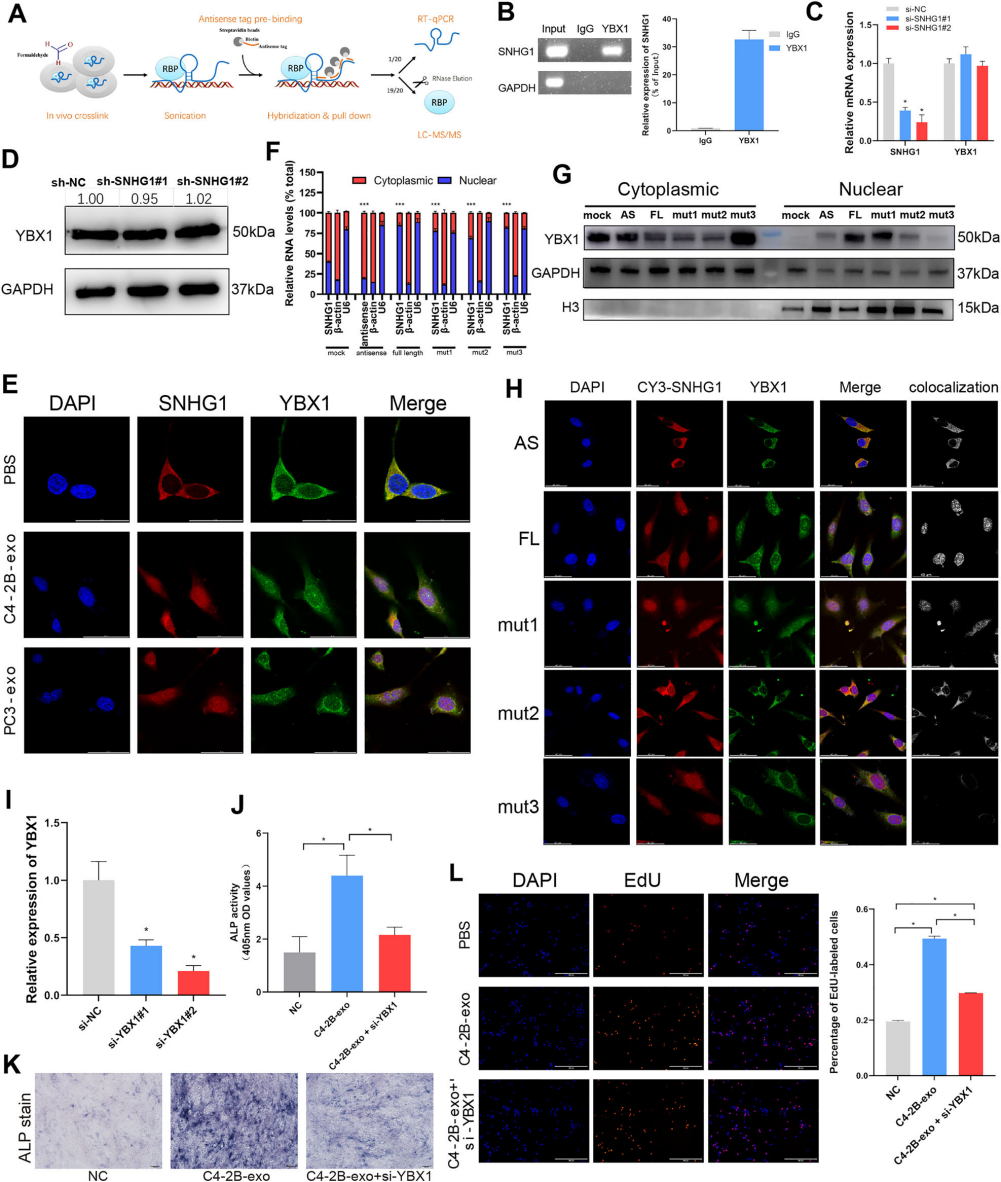
SNHG1 interacts with the transcription factor YBX1 and induces YBX1 into the nucleus. **A** ChIRP analysis of SNHG1-interacting proteins and chromatin in hFOB. Proteins were identified using mass spectrometry, and ChIRP-Sequencing library preparation was performed according to Illumina’s protocol Preparing Samples for ChIRP Sequencing of DNA. **B** RIP-qPCR assay showed SNHG1 can interact with YBX1. **C** SNHG1 or YBX1 expression in hFOB knockdown of SNHG1 measured by RT-qPCR, normalized to GAPDH. **D** Western blotting showed YBX1 level of hFOB after knockdown of *SHNG1*. Protein expression grayscale values were analyzed by imageJ, normalized to GAPDH. **E** FISH combined with IF analysis of SNHG1 RNA and YBX1 protein localization alteration after hFOB treatment with PCa exosomes. The scale bar indicates 50 μm. **F** Expression levels of SNHG1 in subcellular fractions of hFOB cells after co-culture with CY3-SHNG1 mutants were detected by RT-qPCR. U6 and β-actin were used as nuclear and cytoplasmic markers, respectively. U6 or ACTB was used as a loading control in RT-qPCR. **G** Protein levels of YBX1 in subcellular fractions of hFOB cells after co-culture with CY3-SHNG1 mutants were detected by western blot. H3 and GAPDH were used as nuclear and cytoplasmic markers, respectively. **H** Observation of colocalization and subcellular localization between CY3-labeled SNHG1 mutants and YBX1 immunofluorescence staining. Colocalization represents the overlapping signals from CY3 and YBX1 channels. FL: full length; AS: antisense of full length SNHG1; mut3: SNHG1(mut 786–833 region); mut2: SNHG1(mut 636–672 region); mut1: SNHG1(mut 322–462 region). The scale bar indicates 40 μm. **I** YBX1 expression in hFOB of YBX1 interference measured by RT-qPCR, normalized to GAPDH. **J** After treatment with C4-2B exosomes, EdU was assayed for hFOB cells with or without YBX1 interference. The scale bar indicates 400 μm. **K** Detection of ALP activity in hFOB cells with or without YBX1 interference after treatment with C4-2B exosomes. **L** Alkaline phosphatase stain of hFOB with or without YBX1 interference after being treated with C4-2B exosomes. Error bars represent means ± SD, *p < 0.05. ***p < 0.001.

The functions of lncRNAs are reported to be closely related to their subcellular localization and are exerted through interactions with proteins, DNA and other molecules [20]. SNHG1 is more distributed to the nucleus due to the uptake of PCa-derived exosomes by hFOB, and SNHG1 functions as an activator of hFOB. To investigate in which way SNHG1 affects osteoblast activation, we attempted to identify SNHG1-bound proteins and DNA regions in hFOB cells by RNA purification (Chromatin Isolation by RNA Purification, ChIRP) experiment (Fig. 5A). Mass spectrometry identified 35 proteins that specifically bind to SNHG1 (Table S1 and S2; Fig. S2F, G). We assessed protein-RNA-binding strength at LncPro or catRAPID for the top-scoring proteins (QKI, PCBP1, RPL14, PCBP2, YBX1*,* etc.), and YBX1 was the protein with the highest binding propensity score to SNHG1 (Table S3). Among them, YBX1 is a DNA and RNA-binding protein and can act as a transcription factor for its regulatory gene [21]. We selected YBX1 for further validation. In addition, we performed RNA immunoprecipitation (RIP) experiments with anti-YBX1 to show that SNHG1 interacted with YBX1 (Fig. 5B). However, SNHG1 knockdown did not affect the expression level of YBX1 mRNA or protein (Fig. 5C, D), indicating that SNHG1 does not affect YBX1 expression and stability. Since YBX1 is a transcription factor and the uptake of PCa-exosomes alters the subcellular localization of SNHG1 in hFOB. We speculate whether the subcellular localization of YBX1, which interacts with SNHG1, is also altered. FISH assay of SNHG1 combined with immunofluorescence (IF) of YBX1 experiments showed that SNHG1 was co-localized and distributed consistently with YBX1. Moreover, after uptake of PCa-exosomes, as SNHG1 was distributed more into the nucleus, YBX1 was similarly displayed more in the nucleus (Fig. 5E).

To delineate the molecular determinants of SNHG1-dependent YBX1 nuclear translocation, we conducted structural biology predictions and molecular mutation verification. The CatRAPID predicted three high-probability interaction regions on SNHG1 at positions 400, 600, and 850 (Fig. S3A). Combined with AlphaFold3 structural prediction of SNHG1-YBX1 interaction (Fig. S3B), we engineered three CY3-tagged SNHG1 mutants with site-specific antisense substitutions at the predicted YBX1-binding sites: mut1 (786–833), mut2 (636–672), and mut3 (322–462) (Fig. S3C). When transfected into hFOB cells, all mutants exhibited nuclear localization comparable to full-length SNHG1 (Fig. 5F). Strikingly, YBX1 subcellular distribution showed mutation-specific alterations: mut1 retained the strongest nuclear localization of YBX1, while mut2 and mut3 led to a significant reduction of YBX1 in the nucleus, with mut3 inducing nearly complete cytoplasmic retention of YBX1 (Fig. 5G). These findings were confirmed by IF co-staining—YBX1 nuclear signals remained intact in mut1-transfected cells but were markedly diminished in mut2 and mut3 groups (Fig. 5H). Collectively, these data demonstrate that the 636–672 and 322–462 regions of SNHG1 constitute essential interaction domains for SNHG1-dependent nuclear import of YBX1, with the 322–462 region being particularly critical for this regulatory function.

Further, to verify that YBX1 is a co-acting factor in the SNHG1-induced increase in hFOB activity, PCa exosome uptake by hFOB cells using interfering YBX1 (Fig. 5I), ALP assay and EdU proliferation assay showed that interfering with YBX1 rescued part of the SNHG1-induced increase in hFOB activity (Fig. 5J–L). In summary, YBX1 can enter the nucleus by binding SNHG1 and act together to activate hFOB.

### SNHG1 and YBX1 co-regulate MMP16 transcription

**Fig. 6:**
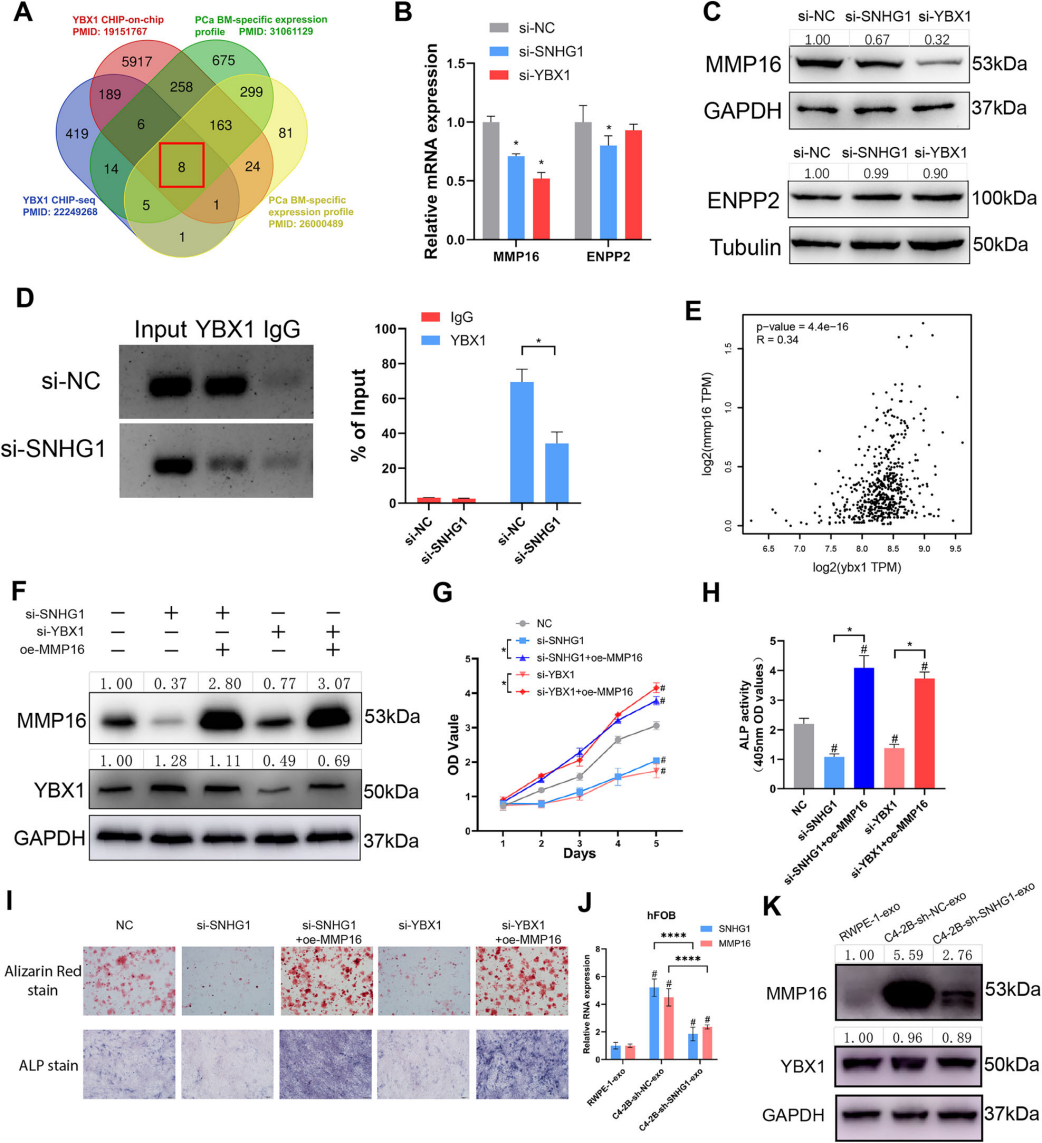
SNHG1 and YBX1 co-regulate MMP16 transcription. **A** Intersection venn diagram of ChIP-seq and ChIP-on-chip data of YBX1 with prostate cancer bone metastasis-related genes. **B** MMP16 or ENPP2 expression in hFOB knockdown of SNHG1 or YBX1 measured by RT-qPCR, normalized to GAPDH. **C** MMP16 or ENPP2 expression in hFOB knockdown of SNHG1 or YBX1 was measured by Western blotting. Protein expression grayscale values were analyzed by ImageJ, normalized to GAPDH or Tubulin. **D** ChIP-qPCR analysis of YBX1 occupancy in the MMP16 promoter after knockdown of SNHG1 in hFOB. **E** The expression correlation between YBX1 and SNHG1 was inferred by bioinformatics (GEPIA 2 (http://gepia2.cancer-pku.cn/)). **F** MMP16 and YBX1 expression in hFOB measured by western blotting. hFOB treated by si-SNHG1 or si-YBX1 with/without oe-MMP16. Protein expression grayscale values were analyzed by imageJ, normalized to GAPDH. **G** CCK8 detects proliferation of hFOBs with knockdown of SNHG1 or YBX1 and overexpression MMP16 or not. **H** Alkaline phosphatase activity or stain of hFOBs with knockdown of SNHG1 or YBX1 and overexpressing MMP16 or not. **I** Alizarin red stain or Alkaline phosphatase stain of hFOB treated with si-SNHG1/si-YBX1 and/or oe-MMP16. **J** MMP16 and SNHG1 expression in hFOB treated with PCa exosomes, measured by RT-qPCR, normalized to GAPDH. **K** MMP16 and YBX1 expression in hFOB treated with PCa exosomes, measured by Western blotting, normalized to GAPDH. Error bars represent means ± SD, ^#^ indicates statistical comparison with the negative control group. ^#^*p* < 0.05, *p < 0.05. ****p < 0.0001.

YBX1 is mainly located in the cytoplasm under normal cellular conditions, and once the YBX1 nuclear translocation occurs, it serves as a transcription factor to regulate downstream gene transcription and participate in the oncogenic processes [22]. Thus, we hypothesized that SNHG1 mediates bone metastasis occurrence by promoting YBX1 nuclear translocation regulation of target downstream genes. For validation, we collected CHIP sequencing and CHIP-on-chip of YBX1 to search for genes that YBX1 may directly bind [23, 24]. Also, we used the cBio Cancer Genomics Portal (http://cbioportal.org: Metastatic Prostate Adenocarcinoma (SU2C/PCF Dream Team, PNAS 2019) [25]; Metastatic PCa (SU2C/PCF Dream Team, Cell 2015) [26]) datasets in order to analyze the specific expression profile of bone metastatic PCa (Table S4). The results showed 8 genes (SELL MMP16 CPVL CHAD ENPP2 COLEC12 KCNK2 RPS27) that may be transcriptionally regulated by YBX1 and associated with PCa bone metastasis (Fig. 6A). Further, we also reviewed CHIRP-seq to identify potential DNA binding sites for SNHG1 in hFOB (Fig. 5A) and focused on the peaks in the promoter region within ±2k around the TSS of the corresponding gene (Table S5). It was found that among the eight genes regulated by YBX1 mentioned above, only the promoter regions of two genes, MMP16 and ENPP2, might also bind SNHG1. When SNHG1 or YBX1 was knocked down in hFOB cells, there was a decrease in the mRNA level of ENPP2 only in si-SNHG1, and no significant difference in the expression of mRNA and protein levels after other interventions, while MMP16 showed a significant decrease in both mRNA and protein levels, confirming the regulatory effect of SNHG1 and YBX1 on MMP16. (Fig. 6B, C). Moreover, the JASPAR (http://jaspar.genereg.net/) predicted sequence binds to the transcription factor YBX1 in the TSS±2k region of MMP16 and ENPP2 at prediction scores >90%, with only two regions (focus only on Watson strand) of MMP16 able to bind to YBX1 (Table S6). Chromatin immunoprecipitation (ChIP) assay followed by qPCR showed that YBX1 was significantly enriched on the MMP16 promoter and the knockdown of SNHG1 resulted in a relative reduction of this enrichment (Fig. 6D). By using the GEPIA (Gene Expression Profile Interaction Analysis) dataset (http://gepia.cancer-pku.cn/), we found that YBX1 was significantly positively correlated with MMP16 in PCa (Fig. 6E).

Then, we proceeded to confirm whether MMP16 contributes to osteoblast activity. Rescue experiments confirmed MMP16 as the essential downstream effector of the SNHG1/YBX1 axis. Strikingly, overexpression of MMP16 not only reversed the impairments in proliferative capacity, ALP activity, and mineralization potential caused by SNHG1 or YBX1 knockdown in hFOB cells, but also further enhanced these osteogenic properties to levels significantly exceeding those of the negative control (NC) group (Fig. 6F–I). Moreover, validation through PCa-derived exosomes demonstrated functional consistency of this molecular axis: after treating hFOB cells with exosomes from RWPE-1, C4-2B-sh-NC, and C4-2B-sh-SNHG1 cell lines, both RNA and protein expression levels of MMP16 correlated positively with exosomal SNHG1 content. Critically, C4-2B-sh-SNHG1 exosome with exosomal SNHG1 rescue confirmed the specific regulation of exosomal SNHG1 on MMP16 expression in hFOB cells (Fig. 6J, K).

Overall, the above results suggest that SNHG1 and YBX1 enter the nucleus to bind the MMP16 promoter region and promote its transcription, which in turn induces increased hFOB activity.

### PCa-derived exosomal SNHG1 induces osteogenesis and promotes PCa bone metastasis in vivo

**Fig. 7:**
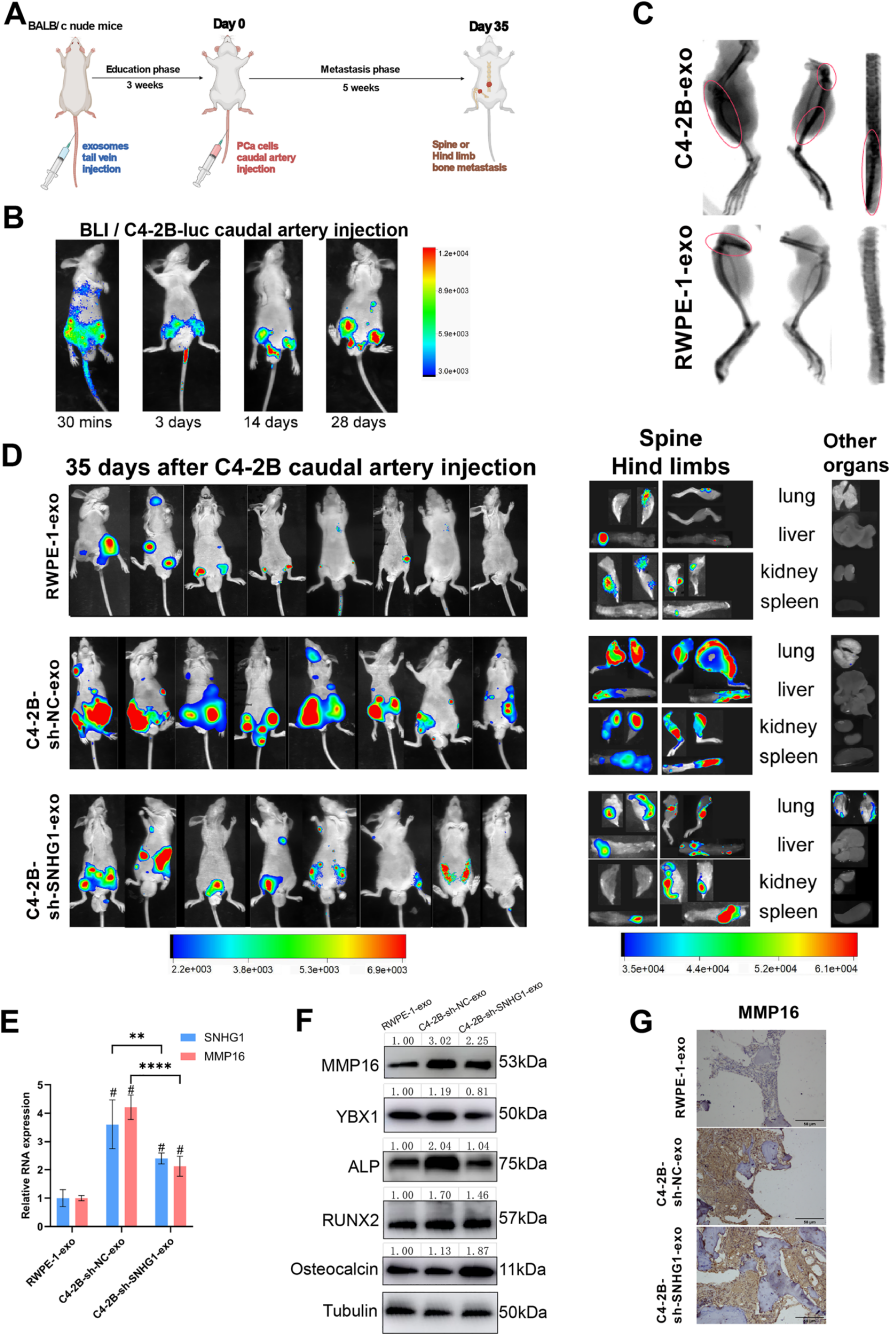
PCa-derived exosomal SNHG1 induced osteogenesis and promoted PCa bone metastasis in vivo. **A** Diagram of the experimental design. Education phase: 3–4 weeks old BABL/c nude mice were first injected with exosomes via the tail vein every 2 days for a total of 3 weeks. Metastasis phase: C4-2B-luciferase cells were injected via the caudal artery to construct a bone metastasis model of prostate cancer. The luciferase activities (radiance values) of bone metastasis were measured by an in vivo imaging system. **B** C4-2B-luciferase cells were injected into the caudal artery to construct a bone metastasis model of prostate cancer, and in vivo imaging was measured at 30 min, 3 days, 14 days, and 28 days after injection, respectively. **C** Bone density of the lower limbs of a bone metastasis model revealed by X-ray. **D** In vivo imaging of bone metastasis model constructs 5 weeks after determining bone metastasis after educating nude mice with exosomes from RWPE-1, with or without SNHG1 knockdown of C4-2B, respectively. Biopsies of spinal bones, hind limbs, and other organs were measured within 2 hours after dissection of these tissues by in vivo imaging system. **E** MMP16 and SNHG1 expression in mouse bone treated with PCa exosomes education, measured by RT-qPCR, normalized to GAPDH. **F** MMP16, YBX1, ALP, RUNX2, Osteocalcin expression in mouse bone treated with PCa exosomes, measured by western blotting, normalized to GAPDH. **G** MMP16 expression in mice PCa bone metastasis with PCa exosomes education pre-treatment, measured by IHC. Error bars represent means ± SD, ^#^indicates statistical comparison with the negative control group. ^#^p < 0.05, **p < 0.01. ****p < 0.0001.

To further evaluate the role of PCa exosome SNHG1 in bone metastasis in vivo, we first gave tail vein administration of exosome education every 2 days for a total of 3 weeks, followed by caudal artery injection of C4-2B cells transfected with luciferase (C4-2B-luc) to construct a bone metastasis model [27] (Fig. 7A). The in vivo distribution and tumorigenesis of C4-2B cells injected via the caudal artery were observed by bioluminescence imaging (BLI) at 30 min, 3 days, 14 days, and 28 days after tumor cell injection (Fig. 7B), showing that caudal artery injection resulted in a more specific distribution of tumor cells to the lower extremities and bone metastasis. X-ray observed that uptake of PCa exosomes after bone metastasis exhibited stronger bone density signal (Fig. 7C). In specific findings, mice educated with C4-2B-sh-NC-exo produced more severe hind limbs bone and spines bone metastasis compared to RWPE-1 exosomes, and administration of C4-2B-sh-SNHG1-exo reduced the extent of this bone metastasis (Fig. 7D). To validate the impact of PCa-derived exosomal SNHG1 on in vivo osteogenic reprogramming, mice were subjected to a 3-week exosome pre-treatment period, followed by extraction of nucleic acids and proteins from bone tissues. Analysis revealed positive correlations between exosomal SNHG1 levels and the induction of MMP16, ALP, and RUNX2. While bone-specific osteocalcin showed discrepancies in rescue experiments, MMP16, ALP, and RUNX2 consistently aligned with exosomal SNHG1 abundance (Fig. 7E, F). Critically, immunohistochemistry (IHC) of PCa bone metastasis specimens confirmed that MMP16 protein expression corresponded precisely to the exosomal SNHG1 gradient across experimental groups: C4-2B-sh-NC > C4-2B-sh-SNHG1 > RWPE-1 (Fig. 7G). The above results suggest that PCa exosome SNHG1 can promote increased bone MMP16 expression and promote the progression of osteogenic nature bone metastasis.

## Discussion

**Fig. 8:**
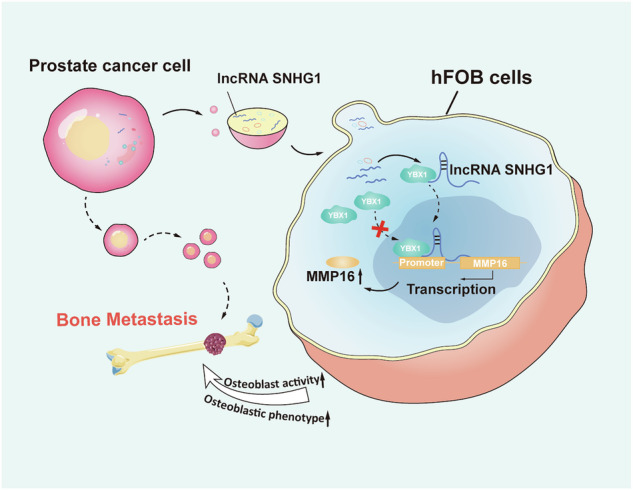
Schematic diagram of the potential mechanism. Schematic representation of the potential molecular mechanism by which exosome SNHG1 promotes PCa bone metastasis.

Bone metastasis occurs in ~70% of patients with advanced PCa and is the leading cause of death in PCa patients [2]. The tumor “seeds” into friendly “soil” and eventually develops into a metastatic lesion [4]. Previous studies revealed that bone is a favorite fertile soil for PCa, where it tends to colonize and proliferate, so attention has turned to the interaction between PCa and the bone microenvironment, mainly including osteoblasts, bone mesenchymal stem cells, and osteoclasts [28]. Tumors can affect the balance between osteoblasts and osteoclasts, disrupting bone homeostasis and altering the skeletal “soil” to create a more favorable microenvironment for the metastasis of multiple primary tumors [29]. Although bone metastasis from breast cancer and other cancers often induces osteolytic lesions, PCa uniquely induces bone formation, and activated osteoblasts are often observed in PCa osteogenic lesions. Tumor activation of osteoblasts is usually the first step in PCa bone metastasis [5]. Therefore, it is important to study the role and mechanism of osteoblasts in PCa bone metastasis. In this study, we show that exosomes derived from PCa shuttle SNHG1 into hFOB, where SNHG1 promotes osteoblast activation by binding YBX1 and inducing its nuclear localization, by activating MMP16 transcription both in vitro and in vivo (Fig. 8).

This study revealed the oncogenic role of lncRNA SNHG1 in PCa bone metastasis. SNHG1 was upregulated in bone metastasis-positive PCa tissues compared to PCa tissues with metastasis from other organs. Moreover, SNHG1 expression was also upregulated in plasma exosomes of patients with PCa bone metastasis as well as in PCa cell line exosomes. LncRNAs are involved in the development of various diseases, and their role in bone metastasis has been demonstrated in many respects. ROR1-HER3 and Hippo-YAP promote osteolytic bone metastasis in breast cancer in an lncRNA MAYA-dependent manner [30]. Wen et al. revealed the mechanism of action of LncRNA SNHG1 regulating PCa bone metastasis via m6A modification [31]. LncRNA SNHG1 has been shown to act as an oncogene in a variety of cancers and to promote tumor metastasis [32]. Interestingly, SNHG1 seems to play a very important role in the development of osteosarcoma through the ceRNA mechanism, suggesting that SNHG1 may have a unique role in the relationship between tumor and bone microenvironment [11, 12, 33]. In addition, the oncogenic role of SNHG1 in the development and progression of PCa has also received attention from various aspects [34–38]. For instance, SNHG1 is stabilized by the protein HnRNPL and activates EMT to promote PCa growth and metastasis [16]. However, these studies have been limited to the role of the tumor cell “seeds” themselves, while the most vital cause of death in PCa, bone metastasis, has not been investigated in depth. Whether SNHG1 can communicate with the tumor and bone microenvironment “soil” is a potential mechanism to reveal the role of PCa bone metastasis. In this study, SNHG1 was distantly transported via the exosomal pathway and taken up by osteoblasts, altering the bone microenvironment to promote osteogenic PCa bone metastasis.

Exosomes secreted by tumors are important mediators of communication between tumor cells and distant organs [39]. In PCa bone metastasis studies, exosomes may alter the bone microenvironment and promote bone metastasis by carrying proteins [40], miRNA [41, 42] or LncRNA [17]. There is a paucity of studies on exosomal lncRNAs in bone metastasis. Exosome protection of long-chain nucleic acids and their ability to act as transport carriers in circulation deserve more attention in PCa bone metastasis studies [43]. Of interest, SNHG1 was highly expressed in plasma samples from lung [14] and liver [15] cancers and has potential as a diagnostic marker for tumors, reflecting the importance of SNHG1 in tumor circulating transport. Recent studies have shown that the exosomal SNHG1 secreted by hypoxic breast cancer induces angiogenesis and promotes tumor growth and metastasis [44]. The exosomal SNHG1 secreted by adipocytes in the multiple myeloma microenvironment protects multiple myeloma cells from chemotherapy-induced apoptosis [45]. We also observed SNHG1 enrichment in plasma exosomes from patients with PCa bone metastasis, as well as in PCa cell exosomes. All these results imply a unique role of exosomal SNHG1 in circulating transport and tumor metastasis in cancer patients and suggest the potential of exosomal SNHG1 as a diagnostic and prognostic marker for PCa bone metastasis.

In addition, we found that exosomal SNHG1 induces YBX1 nuclear localization in hFOB. YBX1 (Y-box binding protein 1), whose name derives from the binding of many genes whose promoter regions are Y-box sequences (5’-CTGATTGG-3’), is a member of the evolutionarily conserved cold shock domain (CSD) protein superfamily [46]. Although YBX1 was originally thought to bind to DNA as a transcription factor for genes, it has been found over the years that YBX1 is involved in almost all DNA- and RNA-related processes and is dependent on its subcellular localization and modification, including DNA repair, RNA-binding, precursor mRNA shearing, and RNA stabilization [21, 47, 48]. YBX1 acts as a transcription factor to activate gene transcription when cells are stressed, such as by cold stimulation, UV radiation or chemotherapy treatment [49]. In cancer, increased expression of YBX1 or increased nuclear distribution ratio was shown to lead to the expression of genes for cell proliferation, differentiation, multi-drug resistance, etc [48]. YBX1 not only regulates the expression of growth-promoting genes such as VEGF [50], EGFR [51], HER2 [52], but also controls genes involved in cell adhesion and motility, such as vimentin and matrix metalloproteinases (MMP2, MMP14) [53–55]. YBX1 is a multifunctional hub in cancer and promotes cancer progression through multiple mechanisms in different cancers [48]. Serum YBX1 can be used as a molecular marker in patients with bone metastases from breast cancer, and serum YBX1-positive patients also have higher expression of IL-6, a known osteogenesis-inducing molecule [56]. In PCa, YBX1 is primarily associated with the regulation of androgen receptor (AR) signaling. YBX1 regulates AR expression at the transcriptional level, and increased nuclear YBX1 signaling was found to be significantly associated with PCa Gleason score and AR expression [57]. A study that analyzed tissue microarrays from 11,152 PCas showed that YBX1 was detectable in 86.3% of PCa, absent or weak relative to normal epithelium, and that YBX1 nuclear staining was strongly associated with poor patient prognosis, tumor stage, Gleason score, and lymph node metastasis [58]. Besides, YBX1 is a key molecule in the progression of PCa to CRPC, which regulates the expression of AR-V7 and can enhance the resistance of enzalutamide [59]. These studies have demonstrated the importance of YBX1, especially its nuclear localization, in the development of cancer. Studies of YBX1 as an RNA-binding protein interacting with lncRNA have also been reported. LncRNA HCP5 helps YBX1 nuclear localization and promotes MSH5 transcription and DNA damage repair in granulosa cells from patients with premature ovarian insufficiency [60]. LINC00857 is upregulated in lung cancer cells by a mechanism that involves LINC00857 binding to YBX1, preventing its proteasomal degradation, increasing its nuclear translocation, and promoting MET expression to regulate biological processes such as cell proliferation [61]. LncRNA BX111 recruits YBX1 to activate ZEB1 expression and induce EMT, promoting metastasis and progression of pancreatic cancer [62]. Coincidentally, the highest SNHG1 binding protein score in neuroblastoma also includes YBX1 (and also HnRNPL and MATR3) [63], where the role of HnRNPL interacting with SNHG1 in PCa has been demonstrated [16]. Our study demonstrates that uptake of PCa exosome SNHG1 by osteoblasts induces YBX1 nuclear translocation—specifically mediated through SNHG1 bases 322-462 and 636-672—leading to increased osteoblast activity and promoting PCa bone metastasis.

The role of MMPs in tumor development and metastasis has been well studied [64], and they also play a key role in the process of bone development [65, 66]. MMP16 and MMP14 were expressed in skeletal mesenchymal tissue and peri-skeletal soft connective tissue, and MMP16-deficient mice showed bone growth inhibition [67]. Osteosarcoma, a tumor with a predominantly altered osteogenic phenotype, with high expression of MMP16, promotes tumor progression and metastasis [68, 69]. YBX1 induces increased expression of MMP14 in breast cancer, and overexpression of YBX1 leads to more locally invasive, lung metastatic and highly lethal breast cancer cells [55]. High expression of MMP16 in PCa is associated with tumor staging and tumor cell metastasis, and its membrane localization is functionally required [70]. In the studies of other contributors [25, 26], we noted high expression of multiple MMP molecules specifically in PCa bone metastasis samples relative to other metastases, including MMP16, MMP13, MMP9, MMP25, etc (Fig. S4), suggesting an important role of MMP molecules in PCa bone metastasis. In this study, we found that SNHG1 and YBX1 could activate the transcription of MMP16 after nuclear entry, where SNHG1 was localized to the promoter region of MMP16 through the principle of base complementary pairing, and YBX1 acted as a transcription factor to initiate the transcription of MMP16.

The level of evidence from animal experiments in tumor metastasis studies is higher. In studies of PCa bone metastasis, previous methods of inducing bone metastasis include intracardiac, caudal vein, intraosseous, and orthotopic injections, but all of these methods have corresponding limitations [71]. In contrast, the caudal artery injection approach, a newly reported method for modeling bone metastasis in recent years, has the advantages of more efficient bone metastasis, low mortality, and simplicity of operation [27]. The reliability and validity of this approach have also been proven in practice in other bone metastasis studies [72, 73]. In our study, we first injected PCa exosomes through the tail vein to simulate the in vivo circulation of exosomes from PCa patients, and then injected PCa cells through the caudal artery to successfully establish a PCa hind limb bone metastasis xenograft model.

Overall, the current study demonstrates that SNHG1 alters the nuclear localization of YBX1 in osteoblasts through PCa exosome transfer, which enhances the osteogenic activity of hFOB by activating MMP16 transcription (Fig. 8). Our findings show new perspectives for the development of effective therapeutic strategies to combat bone metastasis of PCa. The exact process of how PCa secretes SNHG1, the mechanism of nucleation after binding to YBX1, and bone metastasis after induction of osteogenic activity has not been discussed very clearly and needs to be further investigated in future experiments.

## Materials and methods

### PCa samples and clinical information

Plasma samples and tissue samples from PCa patients were collected from Zhujiang Hospital of Southern Medical University in 2015–2021. The diagnosis of PCa bone metastasis is confirmed by an experienced radiologist. Plasma samples were collected before surgery, and tissue samples were collected after surgery and confirmed by an experienced pathologist. The plasma samples were centrifuged to separate the red blood cells and stored at −80 °C, and tissue samples were frozen in liquid nitrogen and then stored at −80 °C. The study design was approved by the Institutional Research Ethics Committee of Southern Medical University before tissue collection. All patients provided written informed consent.

### Cell culture and cell lines

293 T, Human PCa cell lines PC3, DU145, C4-2, C4-2B, 22RV1, human immortalized prostate epithelial cell line RWPE-1, and human SV40 transfected osteoblasts hFOB1.19 (hFOB) were purchased from ATCC. All cell lines were identified by STR profiling. 293 T cells were cultured in DMEM medium (Gibco, USA) with 10% fetal bovine serum (Gibco). PCa cells were cultured in RPMI 1640 medium (Gibco) with 10% fetal bovine serum; RWPE-1 was cultured in keratinocyte serum-free medium (KSFM) (Gibco) with 5 ng/mL EBS. (Gibco); RWPE-1 was cultured in Keratinocyte Serum-Free Medium (KSFM, Gibco) supplemented with 5 ng/mL EGF (epidermal growth factor, Gibco) in a humidified environment with 5% CO_2_ at 37 °C. hFOB were cultured with DMEM/F12 medium (Gibco, USA) supplemented with 10% fetal bovine serum in a humidified environment containing 5% CO_2_ at 34 °C. Serum in the culture environment before collection of exosomes was replaced with 5% exosome-depleted fetal bovine serum (Gibco).

### Exosome experiments

Processing Prior to Extraction of Exosomes from Plasma: 10 mL of whole blood is drawn from the patient, collected in an EDTA anticoagulation tube, and centrifuged at 2000 × *g* for 10 minutes at 4 °C. The supernatant is then centrifuged at 3000 × *g* for 20 minutes at 4 °C. Then, nearly 4 mL of the plasma fraction (upper layer of yellow liquid) is withdrawn, and the supernatant is centrifuged at 3000 × *g* for 20 min at 4 °C. Finally, the supernatant was collected into a new centrifuge tube and stored at −80 °C for subsequent exosome purification. Prior to the collection of cellular exosomes, adherent cells were rinsed twice with PBS and incubated with exosome-depleted FBS medium for 48 h. The cell supernatant was then collected, and the medium was filtered through a 0.22 μm PVDF filter (Millipore, USA).

Exosomes were isolated by differential centrifugation (Beckman, Optima XPN-100): the supernatant was centrifuged at 500 × *g* for 5 min to separate the cells, and then centrifuged at 2000 × *g* for 10 min to separate the remaining cellular debris. The supernatant is centrifuged at 10,000 × *g* for 30 minutes at 4 °C to isolate the large proteins and then centrifuged again at 120,000 × *g* for 70 minutes at 4 °C. The final precipitate was collected as exosomes and stored at −80 °C.

Exosomes were detected by negative staining using an electron microscope and quantified using a NanoSight NS300 instrument (Malvern Instruments Ltd., UK) equipped with NTA 3.0 analysis software (Malvern Instruments Ltd., UK). Exosomes were used for RNA extraction, normalized to exogenous λ polyA (Takara, China) for qPCR; for in vitro exosome treatment, 1 µg of exosomes (equivalent to exosomes collected from ~5 × 10^6^ producer cells) was directly added to 2 × 10^5^ recipient cells. For in vivo exosome treatment, exosomes were injected into the tail vein every 2 days (5 µg exosomes per injection).

After exosome extraction, the samples were taken on a copper mesh, and TEM capture was performed in Building 58 of the Guangdong Academy of Sciences. A suitable field of view was selected for subsequent analysis. The TEM unit was exclusively handled by an expert in charge at the Guangdong Academy of Chinese Medicine, according to the requirements of the unit.

For the exosome uptake assay, we labeled exosomes using the PKH67 Green Fluorescent Cell Linker Mini Kit (Fluorescence, Guangzhou) based on guidelines specified by the manufacturer. Briefly, 2 μg exosomes were resuspended in 500 μL PBS and filtered using a 0.22 μm filter. The exosomes were added to 30% confluent hFOB cells in a confocal dish and cultured for 24 h. Next, cells were fixed for IF staining. Finally, the nuclei of cells were stained using 4’,6-diamidino-2-phenylindole (DAPI, InvitrogenTM, USA).

### Reagents and transfection

SNHG1 short hairpin RNAs (shRNAs) sequence was inserted into Plko.1-puro vector (Addgene). An overexpression plasmid vector was assembled by inserting the full-length cDNA of MMP16 or SNHG1 (ENST00000689147.1) into vector pLVX-MCS-IRES-puro (GeneChem, China).

To package the lentivirus, pLKO.1 or pLVX was co-transfected into 293 T cells (2:1:1 μg) with the lentiviral packaging plasmids psPAX2 and pMD2.G (Addgene). Supernatants were collected after 48 h of co-transfection and filtered through a 0.22 μm PVDF filter.

siRNA (small interfering RNA) was purchased from RiboBio (Guangzhou, China).

Cy3-SNHG1 and Cy3-mutant oligo were purchased from RiboBio. For detailed mutagenesis specifications, refer to the Fig. S3 legend.

Lentiviruses of luciferase-neo were purchased from Genechem (Shanghai, China).

Transfection of plasmids was performed using jetPEI®. Transfection of oligo or siRNA (GenePharma, China) was performed using jetPRIME® at a final concentration of 100 nM. Lentiviruses were mixed with polybrene (Biosharp, China) and transfected. Depending on the resistance type, 2 μg/ml puromycin was screened for 4 days or 200 μg/ml G418 for 7 days.

Sequences of shRNA or siRNA used in this study are listed in Supplemental Methods.

### Western blot

Proteins from cells and exosomes were extracted using RIPA lysis buffer (Biomed, China) containing protease inhibitors, phosphatase inhibitors and PMSF (Solarbio, China). Bone tissue protein extraction was performed using the Minute™ Total Protein Extraction Kit for Bone Tissue (Invent Biotechnologies, USA) following the manufacturer’s protocols. Then, 20–30 μg of proteins were separated on SDS-PAGE and electrotransferred onto a PVDF membrane (Millipore, USA). After BSA blocking, the primary antibody, followed by the secondary antibody, was incubated. Bands were visualized using an enhanced chemiluminescence kit (Pierce, USA), and relative intensities were quantified using ImageJ (National Institutes of Health). Antibodies were listed in Supplemental Methods.

### Fluorescence in situ hybridization (FISH) and IF

Quantification of SNHG1 in PCa clinical samples was determined by expression analysis of SNHG1-positive cells. Briefly, paraffin-embedded sections were deparaffinized and hybridized with Cy3-labeled SNHG1 (Ribobio, China). Nuclei were counterstained with DAPI.

SNHG1 expression and its interrelationship with proteins in exosome-treated hFOB cells were detected in combination with FISH and IF experiments. Briefly, cells were fixed with 4% paraformaldehyde for 10 min at room temperature, rinsed with PBS and permeabilized with 0.2% Triton-X-100 for 15 min. Cells were then blocked with goat serum for 1 hour at room temperature. Afterwards, cells were hybridized with Cy3-labeled SNHG1 probe (Ribobio). Finally, cells were incubated overnight at 4 °C with anti-YBX1 primary antibody and then incubated for 1 hour at room temperature with Alexa Fluor 488-conjugated secondary antibody. The nuclei were then counterstained with DAPI, and the images were visualized with a laser scanning confocal microscope (LSM880, Zeiss).

### RNA isolation, followed by RT-qPCR analysis and Sanger sequencing

Total RNA of cells was extracted by TRIzol® (Invitrogen, USA) according to the manufacturer’s instructions. RNA was extracted from bone using the MolPure Bone RNA Kit (Yeasen Biotechnology, China). Real-time quantitative PCR was performed on triplicate samples in a reaction mix of SYBR Green (Takara, China) with ABI7500 Fast Real-Time PCR System (Applied Biosystems, USA). The mRNA and lncRNA levels were normalized against GAPDH in cell lysates. The RNA levels in culture medium, plasma and exosomes were normalized against a synthesized exogenous reference λ polyA+ RNA (Takara, China) according to the manufacturer’s instructions. The expression of indicated genes was normalized to endogenous or exogenous reference control by using the 2−ΔΔCt method. Sequences of primers used for RT-qPCR in this study are listed in Supplemental Methods. The PCR amplified products were analyzed by Sanger sequencing to validate the target sequences, as shown in Fig. S1E.

### RIP and ChIP

RIP assays were conducted using the EZ-Magna RIP RNA-Binding Protein Immunoprecipitation Kit (Millipore, USA) according to the manufacturer’s instructions. The RNA fraction precipitated by RIP was analyzed by qPCR. ChIP assays were performed using EZ-ChIP Kit (Millipore). Chromatin was immunoprecipitated with the indicated antibodies and analyzed by qPCR. Sequences of primers used for ChIP-qPCR in this study are listed in Supplemental Methods.

### ChChIRP assays

ChIRP experiments were performed using the Magna ChIRP RNA Interactome Kit (Millipore, USA) according to the manufacturer’s protocol. Cells were cross-linked using 1% glutaraldehyde and then sonicated 40 times at 4 °C at high intensity in 15-second pulses and 1-minute intervals. The length of sheared DNA was confirmed by 1% agarose gel electrophoresis analysis. A probe for SNHG1 (100 pmol) was used to hybridize with sonicated cell lysates and streptavidin-coated magnetic beads. DNA isolation was carried out according to standard protocols, and DNA libraries were built. ChIRP-sequencing library preparation was performed according to Illumina’s protocol for DNA ChIRP-sequencing sample preparation. Proteins were collected by cross-linking with 3% formaldehyde, and isolated proteins were washed and heated several times. Proteins were identified by mass spectrometry.

### Cell viability (CCK8)

Cell viability was quantified with Cell Counting kit-8 (Dojindo) following the Producer’s instructions. Finally, a microplate reader (EXL800, BioTek Instruments) was applied to detect the optical density (OD) at 450 nm.

### EdU incorporation assay

EdU cell proliferation was analyzed with Cell-Light EdU Apollo567 In Vitro Kit (Ribobio) following the manufacturer’s instructions. Then, the cells were pictured with a microscope (Olympus, Japan). Finally, the ratio of EdU-stained cells (red fluorescence) to Hoechst-labeled cells (blue fluorescence) in each well was measured.

### Co-culture transwell migration assays

We used Transwell membranes (Corning Costar, USA) with a pore size of 8.0 μm to detect cell migration. The hFOB cells were first grown in the bottom well and then incubated with C4-2B exosomes for 24 hours. After replacing the culture medium, C4-2B cells were planted in the upper chamber of the transwell. 24 hours later, the cells were fixed and stained with 4% PFA and Giemsa (Bost Co., Ltd., Wu, China). Finally, the upper surface of the membrane was wiped, and the cells were imaged using an inverted microscope (DP72, Olympus, Tokyo, Japan). Five fields of view were randomly selected, and cells were counted using “ImageJ” software.

### ALP activity and mineralization assays

For ALP activity, cell lysates were incubated with p-nitrophenylphosphate, and the OD405 was measured. For ALP staining, the culture was fixed with 10% formalin for 1 min and incubated with BCIP/NBT at room temperature for 10 min. For Alizarin staining, the formalin-fixed cells were covered with freshly prepared Alizarin Red S solution and incubated at room temperature in the dark for 60 min.

### Separation of nuclear and cytoplasmic fractions

To detect the distribution of RNA or proteins, cells were first separated into cytoplasmic and nuclear fractions using the NE-PER Nuclear and Cytoplasmic Extraction Kit (ThermoFisher, USA) for protein extraction, or Cytoplasmic & Nuclear RNA Purification Kit (NORGEN, Canada) for RNA extraction. Then, cytoplasmic and nuclear RNA or protein were extracted using Trizol reagent or RIPA. Finally, the lncRNA SNHG1 content in each fraction was determined by RT-qPCR, with U6 as an endogenous gene in the cytosolic fraction. The cytoplasmic fraction used β-actin as the endogenous gene.

### Animal experiments

Three to four-week-old male BALB/C nude mice were provided by the Animal Center of Southern Medical University (Guangzhou, China) and housed under specific pathogen-free conditions. Exosomes were first injected 5 μg/100 μl via the tail vein every 2 days for 3 weeks (Education phase). Then, C4-2B-luciferase 2 × 10^6^ cells were rapidly injected through the caudal artery to construct a PCa bone metastasis model [27, 74]. Thirty-five days after tumor cell injection, D-luciferase potassium salt was injected intraperitoneally., images were captured using a bioluminescence imaging system (in Vivo FX Pro) to assess luciferase signal. Strong fluorescent signals in nude mice indicated sites of tumor metastasis. After mice were sacrificed, spinal bones, hind limbs, and other organs were harvested to detect the bioluminescent signal. Spinal bones and hind limbs were further X-rayed. Animal studies were performed in accordance with institutional guidelines approved by Zhujiang Hospital of Southern Medical University.

### Immunohistochemistry

Bone metastasis specimens were decalcified in 10% EDTA (pH 7.4) for 14 days at 4 °C, paraffin-embedded. IHC was performed using the PV-6000-6.0 kit (Zhongshan Jinqiao Biotechnology, China) following the guidelines specified by the manufacturer. The primary antibodies of MMP16 used were 1:500.

### Computational Prediction of SNHG1-YBX1 Interaction

CatRAPID (http://service.tartaglialab.com/page/catrapid_group) prediction of YBX1-SNHG1 interaction was performed using the CatRAPID Fragments module with default parameters. Molecular docking was conducted via the AlphaFold3 server (https://alphafoldserver.com) using full-length unmodified sequences of YBX1 and SNHG1. The output docking complex was analyzed in PyMOL v3 (Schrödinger, USA).

### Public data analysis

Publicly available PCa tissue data were downloaded from The Cancer Genome Atlas (TCGA). SNHG1 expression and survival curves were analyzed through the Gepia website (http://gepia2.cancer-pku.cn). PCa metastatic samples sequencing data were downloaded from NCBI GEO GSE147250 dataset, DEG difference analysis and heatmaps were performed using morpheus tools website: https://clue.io/morpheus. YBX1 ChIP-seq data were obtained from PMID:22249268 [23]. chip-on-chip data was obtained from PMID:19151767 [24]. Additional data were obtained from cBio Cancer Genomics Portal (http://cbioportal.org).

### Statistical analysis

Statistical analyses of this study were performed using GraphPad Prism8.03 (GraphPad Software, La Jolla, USA). The two-tailed Student’s *t* test was recommended for the data analysis between two groups whose data follow a normal distribution. For data that did not conform to the normal distribution, the Mann–Whitney test was accepted. The log-rank test was used to calculate the Kaplan–Meier survival curve. One-way analysis of variance was utilized to analyze continuous variable groups. Statistical significance was defined as a *P* value <0.05.

## Supplementary information

Figure S1

Figure S2

Figure S3

Figure S4

Figure legends of Supplement Figure S1–4

Table S1

Table S2

Table S3

Table S4

Table S5

Table S6

Supplemental Methods

Original Data of Western blot

## Data Availability

The raw data supporting the results of this study will be made available by the authors, without undue reservation.
