# Supplementary figures and images for: Exosome-transmitted long noncoding RNA SNHG1 promotes prostate cancer bone metastasis via YBX1/MMP16 axis
Source: Cell Death Discov. 2026 Jan 8;12:7. doi: 10.1038/s41420-025-02855-5 (PMC12783806; doi:10.1038/s41420-025-02855-5)

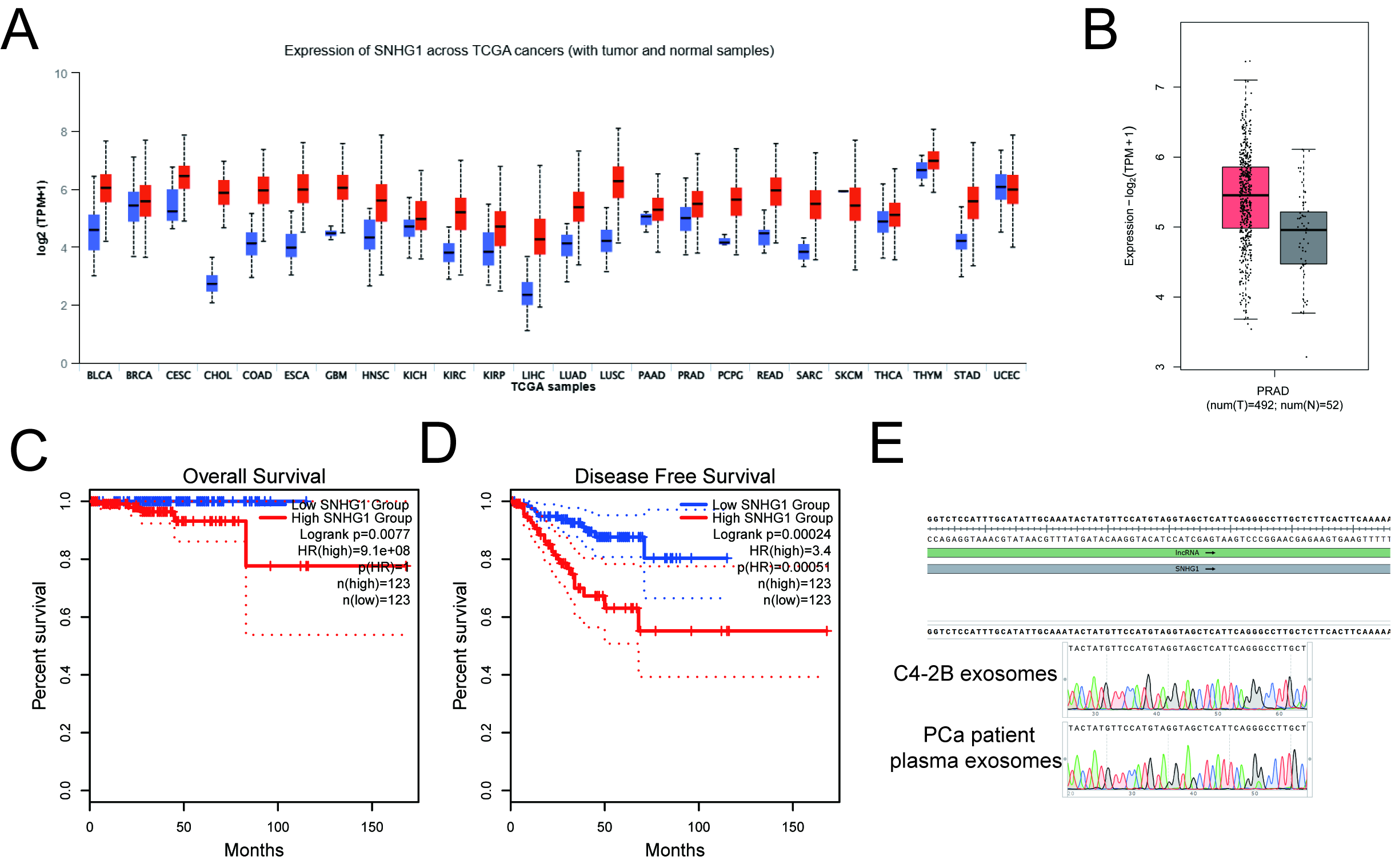

Supplement: Supplementary file 1 — Figure S1 [file 41420_2025_2855_MOESM1_ESM.tif]

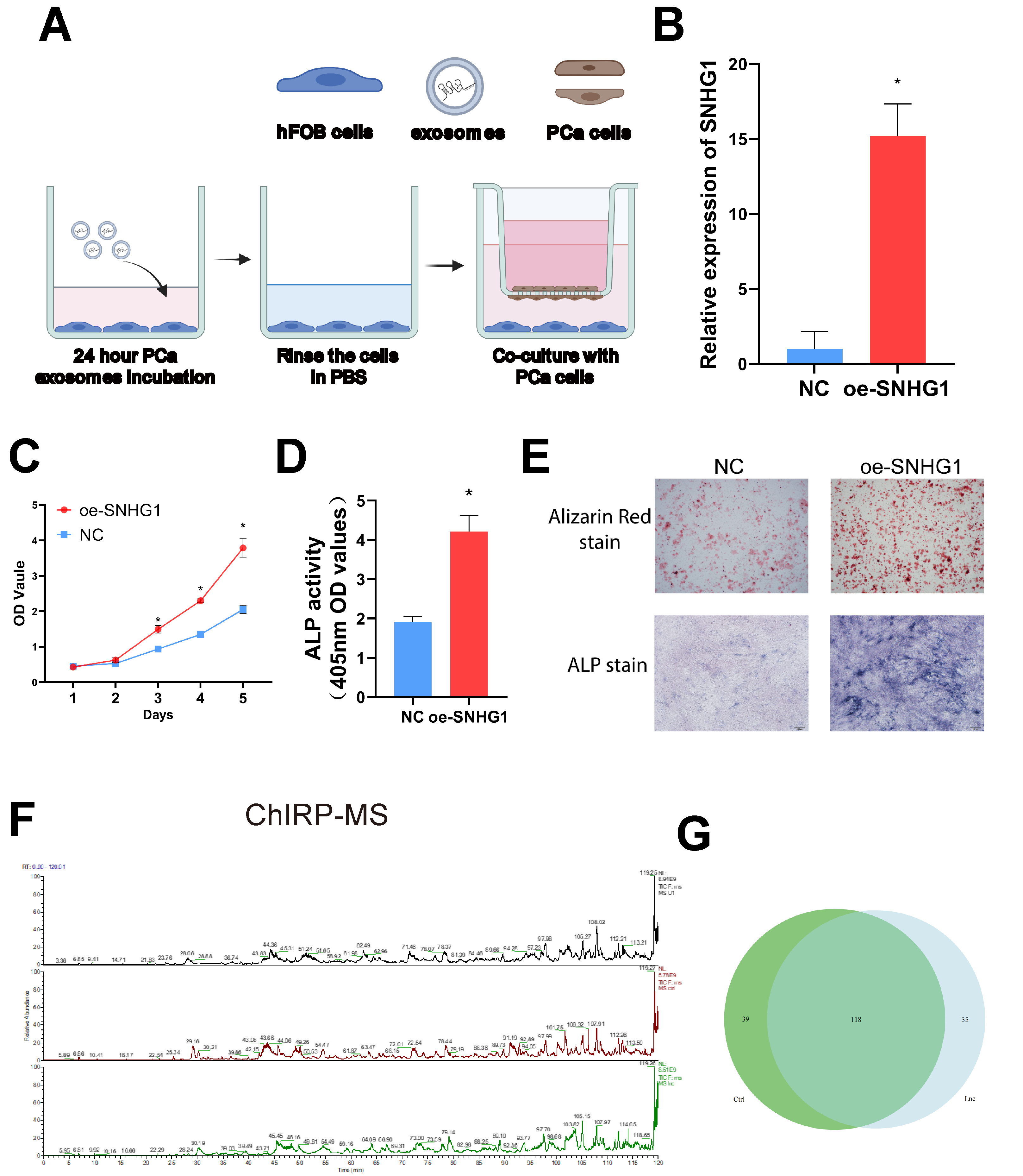

Supplement: Supplementary file 2 — Figure S2 [file 41420_2025_2855_MOESM2_ESM.tif]

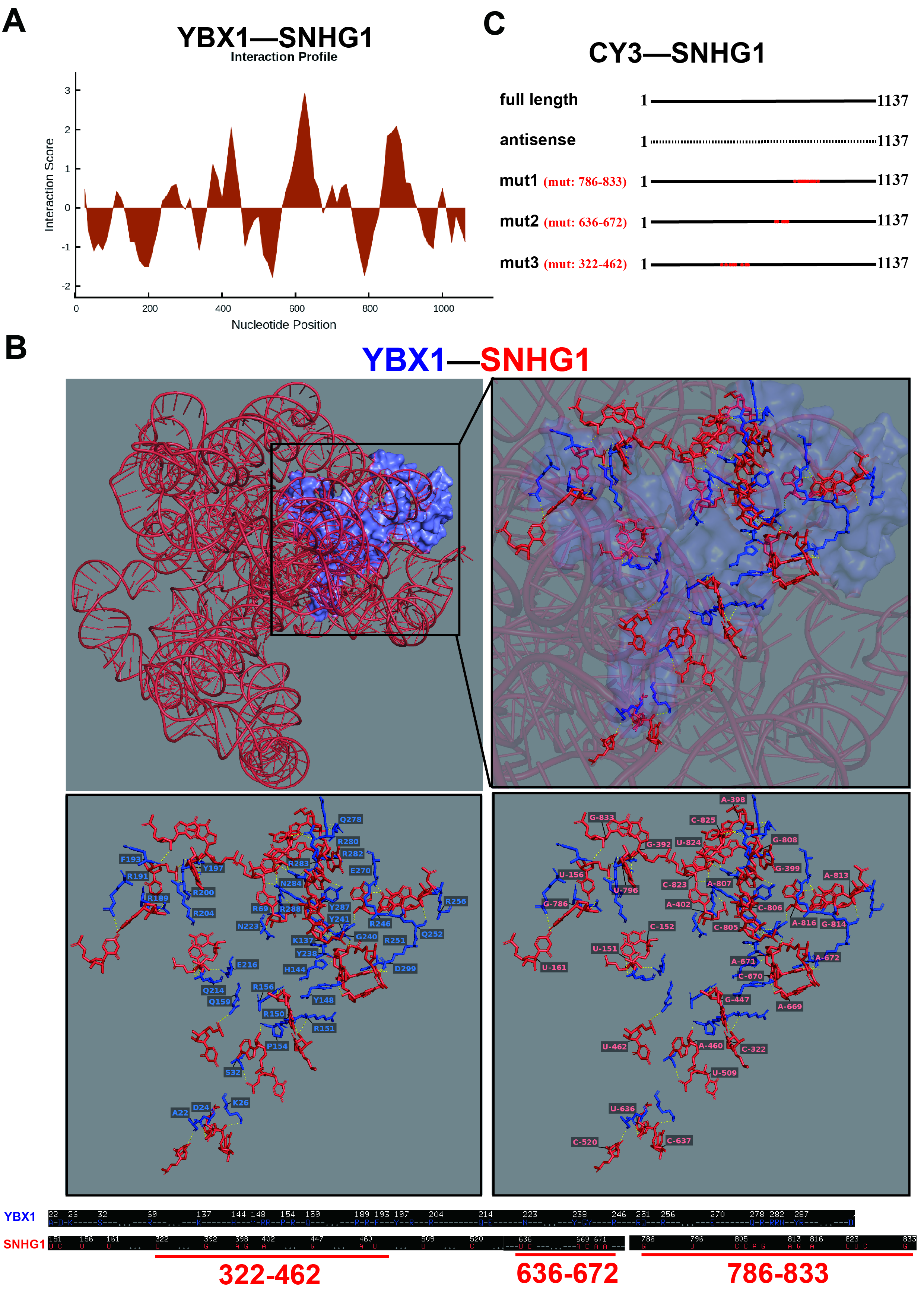

Supplement: Supplementary file 3 — Figure S3 [file 41420_2025_2855_MOESM3_ESM.tif]

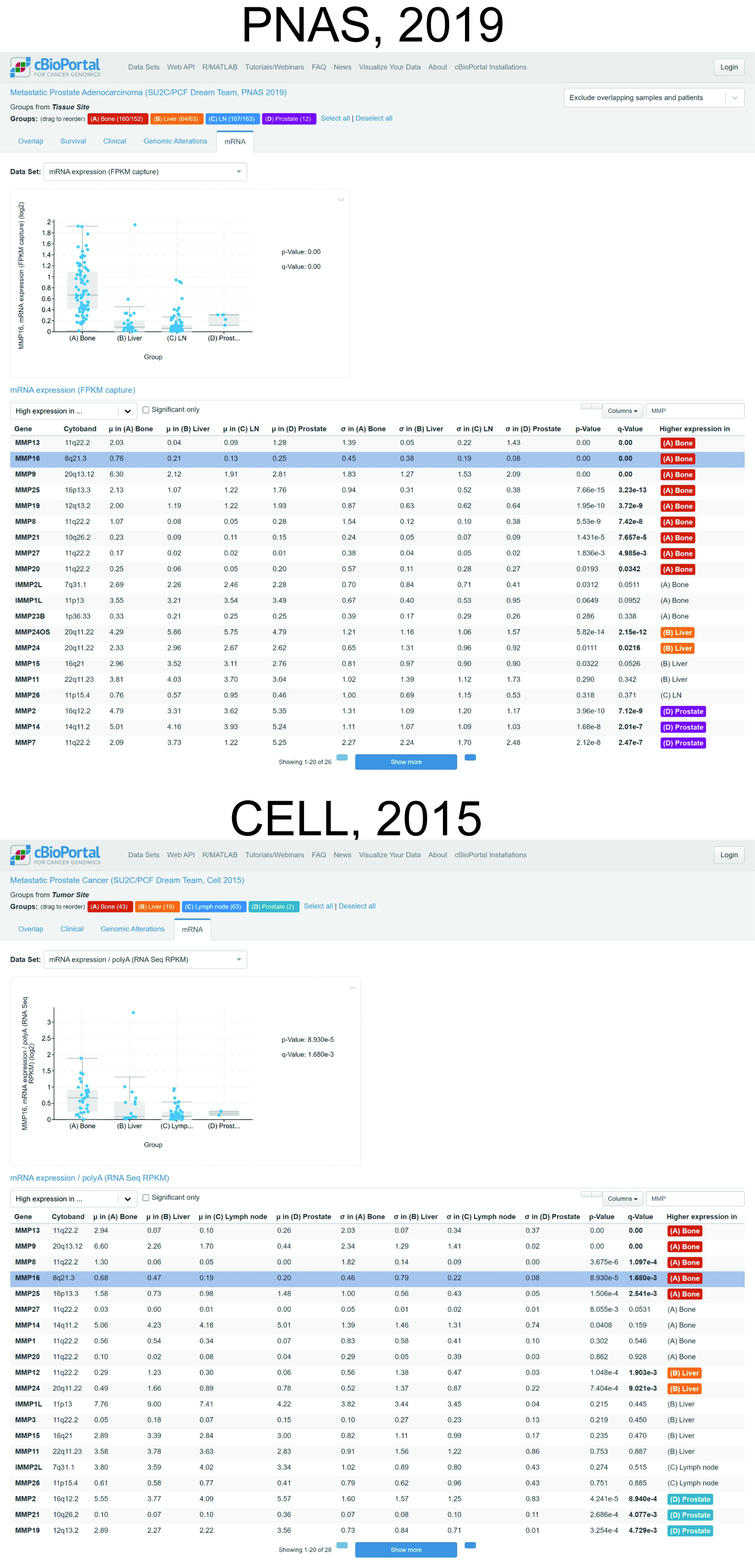

Supplement: Supplementary file 4 — Figure S4 [file 41420_2025_2855_MOESM4_ESM.tif]
