# Supplementary material for: Exosome-transmitted long noncoding RNA SNHG1 promotes prostate cancer bone metastasis via YBX1/MMP16 axis
Source: Cell Death Discov. 2026 Jan 8;12:7. doi: 10.1038/s41420-025-02855-5 (PMC12783806; doi:10.1038/s41420-025-02855-5)
Supplement: Supplementary file 5 — Figure legends of Supplement Figure S1–4 [file 41420_2025_2855_MOESM5_ESM.docx]

**Figure S1.**

(A) SNHG1 pan-cancer expression pattern in TCGA

(B) SNHG1 *expression in prostate cancer carcinoma versus normal tissue in TCGA.*

(C-D) PRAD patients from the TCGA data were divided into low and high SNHG1 expression groups; overall survival (C) and disease-free survival (D) of the patients in the groups used Kaplan-Meier survival analysis. p-values was calculated by the log-rank (Mantel-Cox) test.

(E) Identification of SNHG1 in C4-2B cell exosomes and plasma exosomes from prostate cancer patients by sanger sequencing

**Figure S2.**

(A) Diagram of the experimental design. The bottom well was first planted with hFOB cells and incubated with C4-2B exosomes for 24 hours. After changing the culture medium transwell upper chamber was planted with C4-2B cells. The number of cell migration was detected after 24 hours.

(B) SNHG1 expression in hFOB after overexpression of SNHG1*.*

(C) Detection of alkaline phosphatase (ALP) activity in hFOB after overexpression of SNHG1*.*

(E) Alizarin red stain or Alkaline phosphatase stain of hFOB after overexpression of SNHG1*.*

(F) The ChIRP protein samples were analyzed and identified, and the mass spectrometry results showed the presence of mass peaks.

(G) Venn diagram showing the numbers of SNHG1-specific binding proteins identified by ChIRP-MS.

Error bars represent means ±SD, *p < 0.05.

**Figure S3.**

(A) The CatRAPID fragments module predicts interaction binding regions between SNHG1 and YBX1.

(B) Alphafold 3 molecular docking identifies specific interaction sites on SNHG1 for YBX1:

​322-462 nt: C322, G392, A398, G399, A402, G477, A460, U462

​636-672 nt: U636, C637, A669, C670, A671, A672

​786-833 nt: G786, U796, C805, C806, A807, G808, A813, G814, A816, C823, U824, C825, G833

(C) CY3-labeled SNHG1 mutant constructs include:

FL: Full-length wild-type

AS: Full-length antisense strand (negative control)

Mutants: Complementary base substitutions at all nucleotides illustrated in Panel B: mut1 (786-833 region, 8 nucleotides) ; mut2 (636-672 region 6 nucleotides) ; mut3 (322-462 region 13 nucleotides).

**Figure S4.**

Multiple MMPs were highly expressed in prostate cancer bone metastasis samples relative to other metastases from prostate cancer, *p* < 0.001 (PMID:26000489, PMID:31061129). Data from cBio Cancer Genomics Portal (<http://cbioportal.org>)
