# Supplementary material for: Exosome-transmitted long noncoding RNA SNHG1 promotes prostate cancer bone metastasis via YBX1/MMP16 axis
Source: Cell Death Discov. 2026 Jan 8;12:7. doi: 10.1038/s41420-025-02855-5 (PMC12783806; doi:10.1038/s41420-025-02855-5)
Supplement: Supplementary file 7 — Table S2 [file 41420_2025_2855_MOESM7_ESM.docx]

**Table S2: Significant proteins annotation**

| Symbol | #Unique | FC | Lnc | Ctrl Annotation |
| --- | --- | --- | --- | --- |

| QKI | 14 | 5.61 | 39 | 0 | Protein quaking; RNA-binding protein that plays a central role in myelinization. Binds to the  5’-NACUAAY-N(1,20)- UAAY-3’ RNA core sequence. Regulates target mRNA stability. In addition, acts by regulating pre-mRNA splicing, mRNA export and  protein translation. Required to protect and promote stability of mRNAs such as MBP and CDKN1B.  Regulator of oligodendrocyte differentiation and  maturation in the brain that may play a role in myelin and oligodendrocyte dysfunction in schizophrenia.  Participates in mRNA transport by regulating the  nuclear export of MBP mRNA. Also involved in regulat [...] |
| --- | --- | --- | --- | --- | --- |
| PCBP1 | 4 | 3.44 | 8 | 0 | Poly(rC)-binding protein 1; Single-stranded nucleic  acid binding protein that binds preferentially to oligo dC. In case of infection by poliovirus, plays a role in initiation of viral RNA replication in concert with the viral protein 3CD |
| RPL14 | 2 | 2.57 | 4 | 0 | 60S ribosomal protein L14; Component of the large  ribosomal subunit; Belongs to the eukaryotic ribosomal protein eL14 family |
| PCBP2 | 3 | 2.57 | 4 | 0 | Poly(rC)-binding protein 2; Single-stranded nucleic  acid binding protein that binds preferentially to oligo  dC. Major cellular poly(rC)-binding protein. Binds also  poly(rU) . Negatively regulates cellular antiviral  responses mediated by MAVS signaling. It acts as an adapter between MAVS and the E3 ubiquitin ligase  ITCH, therefore triggering MAVS ubiquitination and degradation |
| YBX1 | 5 | 2.07 | 6 | 1 | Nuclease-sensitive element-binding protein 1; Mediates pre-mRNA alternative splicing regulation. Binds to  splice sites in pre-mRNA and regulates splice site selection. Binds and stabilizes cytoplasmic mRNA. Contributes to the regulation of translation by  modulating the interaction between the mRNA and  eukaryotic initiation factors (By similarity) . Regulates the transcription of numerous genes. Its transcriptional activity on the multidrug resistance gene MDR1 is  enhanced in presence of the APEX1 acetylated form at ’Lys-6’ and ’Lys-7’. Binds to promoters that contain a Y-box (5’-CTGATTG [...] |
| HNRNPK | 12 | 1.85 | 8 | 2 | Heterogeneous nuclear ribonucleoprotein K; One of the major pre-mRNA-binding proteins. Binds tenaciously to poly(C) sequences. Likely to play a role in the  nuclear metabolism of hnRNAs, particularly for  pre-mRNAs that contain cytidine-rich sequences. Can also bind poly(C) single- stranded DNA. Plays an  important role in p53/TP53 response to DNA damage, acting at the level of both transcription activation and repression. When sumoylated, acts as a transcriptional coactivator of p53/TP53, playing a role in  p21/CDKN1A and 14-3-3 sigma/SFN induction (By similarity) . As far as transcription [...] |
| HNRNPAB | 3 | 1.79 | 2 | 0 | Heterogeneous nuclear ribonucleoprotein A/B; Binds single-stranded RNA. Has a high affinity for G- rich  and U-rich regions of hnRNA. Also binds to APOB  mRNA transcripts around the RNA editing site; RNA binding motif containing |
| RPL27A | 2 | 1.79 | 2 | 0 | Ribosomal protein L27a; Belongs to the universal ribosomal protein uL15 family |

HNRNPA3 9 1.79 2 0 Heterogeneous nuclear ribonucleoprotein A3; Plays a

role in cytoplasmic trafficking of RNA. Binds to the

cis-acting response element, A2RE. May be involved in pre-mRNA splicing; RNA binding motif containing

| KRT16 | 11 | 1.75 | 21 | 7 | Keratin, type I cytoskeletal 16; Epidermis-specific type I keratin that plays a key role in skin. Acts as a  regulator of innate immunity in response to skin barrier breach: required for some inflammatory checkpoint for the skin barrier maintenance |
| --- | --- | --- | --- | --- | --- |
| NCL | 6 | 1.57 | 4 | 1 | Nucleolin; Nucleolin is the major nucleolar protein of  growing eukaryotic cells. It is found associated with  intranucleolar chromatin and pre-ribosomal particles.  It induces chromatin decondensation by binding to  histone H1 . It is thought to play a role in pre-rRNA  transcription and ribosome assembly. May play a role  in the process of transcriptional elongation. Binds RNA oligonucleotides with 5’-UUAGGG-3’ repeats more  tightly than the telomeric single-stranded DNA  5’-TTAGGG-3’ repeats |
| RPS10 | 3 | 1.57 | 4 | 1 | 40S ribosomal protein S10; Component of the 40S  ribosomal subunit; Belongs to the eukaryotic ribosomal protein eS10 family |
| HSPE1 | 3 | 1.57 | 4 | 1 | 10 kDa heat shock protein, mitochondrial;  Co-chaperonin implicated in mitochondrial protein  import and macromolecular assembly. Together with  Hsp60, facilitates the correct folding of imported  proteins. May also prevent misfolding and promote the refolding and proper assembly of unfolded polypeptides generated under stress conditions in the mitochondrial matrix. The functional units of these chaperonins  consist of heptameric rings of the large subunit Hsp60,  which function as a back-to-back double ring. In a cyclic reaction, Hsp60 ring complexes bind one  unfolded substrate protein per [...] |
| HIST1H4A | 5 | 1.54 | 11 | 4 | Histone cluster 1 H4 family member f; Core component of nucleosome. Nucleosomes wrap and compact DNA  into chromatin, limiting DNA accessibility to the  cellular machineries which require DNA as a template. Histones thereby play a central role in transcription  regulation, DNA repair, DNA replication and  chromosomal stability. DNA accessibility is regulated  via a complex set of post-translational modifications of histones, also called histone code, and nucleosome  remodeling (By similarity) |
| HIST1H4A | 5 | 1.54 | 11 | 4 | Histone cluster 1 H4 family member k; Core component of nucleosome. Nucleosomes wrap and compact DNA  into chromatin, limiting DNA accessibility to the  cellular machineries which require DNA as a template. Histones thereby play a central role in transcription  regulation, DNA repair, DNA replication and  chromosomal stability. DNA accessibility is regulated  via a complex set of post-translational modifications of histones, also called histone code, and nucleosome  remodeling |
| HIST1H4A | 5 | 1.54 | 11 | 4 | Histone cluster 1 H4 family member d; Core component of nucleosome. Nucleosomes wrap and compact DNA  into chromatin, limiting DNA accessibility to the  cellular machineries which require DNA as a template. Histones thereby play a central role in transcription  regulation, DNA repair, DNA replication and  chromosomal stability. DNA accessibility is regulated  via a complex set of post-translational modifications of histones, also called histone code, and nucleosome  remodeling |
| HIST1H4A | 5 | 1.54 | 11 | 4 | Histone cluster 1 H4 family member i; Core component of nucleosome. Nucleosomes wrap and compact DNA  into chromatin, limiting DNA accessibility to the  cellular machineries which require DNA as a template. Histones thereby play a central role in transcription  regulation, DNA repair, DNA replication and  chromosomal stability. DNA accessibility is regulated  via a complex set of post-translational modifications of histones, also called histone code, and nucleosome  remodeling |

| HIST1H4A | 5 | 1.54 | 11 | 4 | Histone cluster 1 H4 family member j; Core component of nucleosome. Nucleosomes wrap and compact DNA  into chromatin, limiting DNA accessibility to the  cellular machineries which require DNA as a template. Histones thereby play a central role in transcription  regulation, DNA repair, DNA replication and  chromosomal stability. DNA accessibility is regulated  via a complex set of post-translational modifications of histones, also called histone code, and nucleosome  remodeling |
| --- | --- | --- | --- | --- | --- |
| HIST1H4A | 5 | 1.54 | 11 | 4 | Histone cluster 2 H4 family member a; Core component of nucleosome. Nucleosomes wrap and compact DNA  into chromatin, limiting DNA accessibility to the  cellular machineries which require DNA as a template. Histones thereby play a central role in transcription  regulation, DNA repair, DNA replication and  chromosomal stability. DNA accessibility is regulated  via a complex set of post-translational modifications of histones, also called histone code, and nucleosome  remodeling |
| HIST1H4A | 5 | 1.54 | 11 | 4 | Histone cluster 1 H4 family member a; Core component of nucleosome. Nucleosomes wrap and compact DNA  into chromatin, limiting DNA accessibility to the  cellular machineries which require DNA as a template. Histones thereby play a central role in transcription  regulation, DNA repair, DNA replication and  chromosomal stability. DNA accessibility is regulated  via a complex set of post-translational modifications of histones, also called histone code, and nucleosome  remodeling |
| HIST1H4A | 5 | 1.54 | 11 | 4 | Histone cluster 1 H4 family member b; Core component of nucleosome. Nucleosomes wrap and compact DNA  into chromatin, limiting DNA accessibility to the  cellular machineries which require DNA as a template. Histones thereby play a central role in transcription  regulation, DNA repair, DNA replication and  chromosomal stability. DNA accessibility is regulated  via a complex set of post-translational modifications of histones, also called histone code, and nucleosome  remodeling |
| HIST1H4A | 5 | 1.54 | 11 | 4 | Histone H4; Core component of nucleosome.  Nucleosomes wrap and compact DNA into chromatin, limiting DNA accessibility to the cellular machineries which require DNA as a template. Histones thereby  play a central role in transcription regulation, DNA repair, DNA replication and chromosomal stability.  DNA accessibility is regulated via a complex set of  post-translational modifications of histones, also called histone code, and nucleosome remodeling |
| HIST1H4A | 5 | 1.54 | 11 | 4 | Histone cluster 4, H4; Core component of nucleosome. Nucleosomes wrap and compact DNA into chromatin, limiting DNA accessibility to the cellular machineries which require DNA as a template. Histones thereby  play a central role in transcription regulation, DNA  repair, DNA replication and chromosomal stability.  DNA accessibility is regulated via a complex set of  post-translational modifications of histones, also called histone code, and nucleosome remodeling |
| HIST1H4A | 5 | 1.54 | 11 | 4 | Histone cluster 1 H4 family member l; Core component of nucleosome. Nucleosomes wrap and compact DNA  into chromatin, limiting DNA accessibility to the  cellular machineries which require DNA as a template. Histones thereby play a central role in transcription  regulation, DNA repair, DNA replication and  chromosomal stability. DNA accessibility is regulated  via a complex set of post-translational modifications of histones, also called histone code, and nucleosome  remodeling |

| HIST1H4A | 5 | 1.54 | 11 | 4 | Histone cluster 1 H4 family member c; Core component of nucleosome. Nucleosomes wrap and compact DNA  into chromatin, limiting DNA accessibility to the  cellular machineries which require DNA as a template. Histones thereby play a central role in transcription  regulation, DNA repair, DNA replication and  chromosomal stability. DNA accessibility is regulated  via a complex set of post-translational modifications of histones, also called histone code, and nucleosome  remodeling |
| --- | --- | --- | --- | --- | --- |
| HIST1H4A | 5 | 1.54 | 11 | 4 | Histone cluster 1 H4 family member h; Core component of nucleosome. Nucleosomes wrap and compact DNA  into chromatin, limiting DNA accessibility to the  cellular machineries which require DNA as a template. Histones thereby play a central role in transcription  regulation, DNA repair, DNA replication and  chromosomal stability. DNA accessibility is regulated  via a complex set of post-translational modifications of histones, also called histone code, and nucleosome  remodeling |
| HIST1H4A | 5 | 1.54 | 11 | 4 | Histone cluster 2 H4 family member b; Core component of nucleosome. Nucleosomes wrap and compact DNA  into chromatin, limiting DNA accessibility to the  cellular machineries which require DNA as a template. Histones thereby play a central role in transcription  regulation, DNA repair, DNA replication and  chromosomal stability. DNA accessibility is regulated  via a complex set of post-translational modifications of histones, also called histone code, and nucleosome  remodeling |
| RPL13 | 5 | 1.39 | 12 | 5 | Ribosomal protein L13 |
| RPS9 | 7 | 1.38 | 14 | 6 | Ribosomal protein S9 |
| RPS3 | 5 | 1.27 | 9 | 4 | 40S ribosomal protein S3; Involved in translation as a component of the 40S small ribosomal subunit. Has  endonuclease activity and plays a role in repair of  damaged DNA. Cleaves phosphodiester bonds of DNAs containing altered bases with broad specificity and  cleaves supercoiled DNA more efficiently than relaxed DNA. Displays high binding affinity for  7,8-dihydro-8-oxoguanine (8-oxoG), a common DNA  lesion caused by reactive oxygen species (ROS) . Has  also been shown to bind with similar affinity to intact and damaged DNA. Stimulates the N-glycosylase  activity of the base excision protein [...] |
| UBA52 | 2 | 1.25 | 5 | 2 | Ubiquitin-60S ribosomal protein L40; Ubiquitin: Exists either covalently attached to another protein, or free  (unanchored) . When covalently bound, it is conjugated to target proteins via an isopeptide bond either as a  monomer (monoubiquitin), a polymer linked via  different Lys residues of the ubiquitin (polyubiquitin  chains) or a linear polymer linked via the initiator Met of the ubiquitin (linear polyubiquitin chains) .  Polyubiquitin chains, when attached to a target  protein, have different functions depending on the Lys residue of the ubiquitin that is linked: Lys-6-linked  may be invo [...] |
| RPL13A | 3 | 1.25 | 5 | 2 | 60S ribosomal protein L13a; Associated with ribosomes but is not required for canonical ribosome function and has extra-ribosomal functions. Component of the GAIT (gamma interferon-activated inhibitor of translation)  complex which mediates interferon-gamma-induced transcript-selective translation inhibition in  inflammation processes . Upon interferon-gamma  activation and subsequent phosphorylation dissociates  from the ribosome and assembles into the GAIT  complex which binds to stem loop-containing GAIT elements in the 3’-UTR of diverse inflammatory  mRNAs (such as ceruplasmin) and suppre [...] |
| HRNR | 8 | 1.13 | 17 | 9 | Hornerin; Component of the epidermal cornified cell envelopes; Belongs to the S100-fused protein family |
| PKP1 | 5 | 1.07 | 6 | 3 | Plakophilin-1; Seems to play a role in junctional  plaques. Contributes to epidermal morphogenesis; Armadillo repeat containing |

| RPL18 | 6 | 0.91 | 16 | 10 | 60S ribosomal protein L18; Component of the large  ribosomal subunit; Belongs to the eukaryotic ribosomal protein eL18 family |
| --- | --- | --- | --- | --- | --- |
| RPSA | 6 | 0.85 | 8 | 5 | 40S ribosomal protein SA; Required for the assembly  and/or stability of the 40S ribosomal subunit. Required  for the processing of the 20S rRNA- precursor to  mature 18S rRNA in a late step of the maturation of  40S ribosomal subunits. Also functions as a cell surface receptor for laminin. Plays a role in cell adhesion to the basement membrane and in the consequent activation  of signaling transduction pathways. May play a role in cell fate determination and tissue morphogenesis. Acts as a PPP1R16B-dependent substrate of PPP1CA |
| HNRNPA2B1 | 11 | 0.84 | 5 | 3 | Heterogeneous nuclear ribonucleoproteins A2/B1;  Heterogeneous nuclear ribonucleoprotein (hnRNP) that associates with nascent pre-mRNAs, packaging them  into hnRNP particles. The hnRNP particle  arrangement on nascent hnRNA is non- random and sequence-dependent and serves to condense and  stabilize the transcripts and minimize tangling and  knotting. Packaging plays a role in various processes  such as transcription, pre-mRNA processing, RNA  nuclear export, subcellular location, mRNA translation and stability of mature mRNAs. Forms hnRNP  particles with at least 20 other different hnRNP and h [...] |
| AZGP1 | 4 | 0.84 | 5 | 3 | Zinc-alpha-2-glycoprotein; Stimulates lipid degradation in adipocytes and causes the extensive fat losses  associated with some advanced cancers . May bind  polyunsaturated fatty acids; Belongs to the MHC class I family |
| RPL32 | 3 | 0.79 | 2 | 1 | Ribosomal protein L32; Belongs to the eukaryotic ribosomal protein eL32 family |
| RPL28 | 4 | 0.79 | 2 | 1 | 60S ribosomal protein L28; Component of the large  ribosomal subunit; Belongs to the eukaryotic ribosomal protein eL28 family |
| ALOX12B | 2 | 0.79 | 2 | 1 | Arachidonate 12-lipoxygenase, 12R-type; Non-heme iron-containing dioxygenase that catalyzes the  stereo-specific peroxidation of free and esterified  polyunsaturated fatty acids generating a spectrum of bioactive lipid mediators. Mainly converts arachidonic acid to (12R)- hydroperoxyeicosatetraenoic  acid/(12R)-HPETE and minor stereoisomers. In the  skin, acts upstream of ALOXE3 on the lineolate moiety of esterified omega-hydroxyacyl-sphingosine (EOS)  ceramides to produce an epoxy-ketone derivative, a crucial step in the conjugation of  omega-hydroxyceramide to membrane proteins. Therefore [...] |
| P4HB | 2 | 0.79 | 2 | 1 | Protein disulfide-isomerase; This multifunctional protein catalyzes the formation, breakage and  rearrangement of disulfide bonds. At the cell surface,  seems to act as a reductase that cleaves disulfide bonds of proteins attached to the cell. May therefore cause  structural modifications of exofacial proteins. Inside  the cell, seems to form/rearrange disulfide bonds of  nascent proteins. At high concentrations, functions as a chaperone that inhibits aggregation of misfolded  proteins. At low concentrations, facilitates aggregation (anti-chaperone activity) . May be involved with other chape [...] |
| TXN | 2 | 0.79 | 2 | 1 | Thioredoxin; Participates in various redox reactions through the reversible oxidation of its active center dithiol to a disulfide and catalyzes dithiol-disulfide exchange reactions. Plays a role in the reversible  S-nitrosylation of cysteine residues in target proteins,  and thereby contributes to the response to intracellular nitric oxide. Nitrosylates the active site Cys of CASP3 in response to nitric oxide (NO), and thereby inhibits  caspase-3 activity. Induces the FOS/JUN AP-1  DNA-binding activity in ionizing radiation (IR) cells through its oxidation/reduction status and stimulates A [...] |
| RPL4 | 3 | 0.79 | 2 | 1 | L ribosomal proteins |

HIST1H2BN 6 0.75 6 4 Histone H2B type 1-M; Core component of nucleosome.

Nucleosomes wrap and compact DNA into chromatin, limiting DNA accessibility to the cellular machineries which require DNA as a template. Histones thereby

play a central role in transcription regulation, DNA

repair, DNA replication and chromosomal stability.

DNA accessibility is regulated via a complex set of

post-translational modifications of histones, also called histone code, and nucleosome remodeling

DSC3 2 0.65 3 2 Desmocollin-3; Component of intercellular desmosome

junctions. Involved in the interaction of plaque proteins and intermediate filaments mediating cell-cell adhesion. May contribute to epidermal cell positioning

(stratification) by mediating differential adhesiveness between cells that express different isoforms;

Desmosomal cadherins

RPL36 2 0.65 3 2 60S ribosomal protein L36; Component of the large

ribosomal subunit

KRT14 8 0.62 48 38 Keratin, type I cytoskeletal 14; The nonhelical tail

domain is involved in promoting KRT5-KRT14

filaments to self-organize into large bundles and

enhances the mechanical properties involved in

resilience of keratin intermediate filaments in vitro
