# Supplementary material for: Exosome-transmitted long noncoding RNA SNHG1 promotes prostate cancer bone metastasis via YBX1/MMP16 axis
Source: Cell Death Discov. 2026 Jan 8;12:7. doi: 10.1038/s41420-025-02855-5 (PMC12783806; doi:10.1038/s41420-025-02855-5)
Supplement: Supplementary file 11 — Table S6 [file 41420_2025_2855_MOESM11_ESM.docx]

**Table S6**

**ENPP2 TSS ±2k NC_000008.11:c119675404-119671404**

| Matrix ID | Name | Score | Relative score | Sequence ID | Start | End | Strand | Predicted sequence |
| --- | --- | --- | --- | --- | --- | --- | --- | --- |
| UN0139.1 | UN0139.1.YBX1 | 8.73667 | 0.917034158 | NC_000008.11:c119675404-119673404 | 135 | 143 | - | TGTTCCTTC |
| UN0139.1 | UN0139.1.YBX1 | 9.963544 | 0.937566108 | NC_000008.11:c119675404-119673404 | 1450 | 1458 | - | TGCCCCATC |
| UN0139.1 | UN0139.1.YBX1 | 7.899058 | 0.903016585 | NC_000008.11:c119673404-119671404 | 1053 | 1061 | - | CTCTCCATC |

**MMP16 TSS ±2k NC_000008.11:c88329483-88325483**

| Matrix ID | Name | Score | Relative score | Sequence ID | Start | End | Strand | Predicted sequence |
| --- | --- | --- | --- | --- | --- | --- | --- | --- |
| UN0139.1 | UN0139.1.YBX1 | 9.943429 | 0.937229481 | NC_000008.11:c88329483-88327483 | 371 | 379 | + | TTTTCCATC |
| UN0139.1 | UN0139.1.YBX1 | 8.607295 | 0.914869054 | NC_000008.11:c88329483-88327483 | 65 | 73 | - | CACACCATC |
| UN0139.1 | UN0139.1.YBX1 | 8.011803 | 0.904903389 | NC_000008.11:c88329483-88327483 | 149 | 157 | + | TATTACATC |
| UN0139.1 | UN0139.1.YBX1 | 8.704524 | 0.916496198 | NC_000008.11:c88327483-88325483 | 56 | 64 | - | TTCACCATC |
| UN0139.1 | UN0139.1.YBX1 | 7.772162 | 0.900892953 | NC_000008.11:c88327483-88325483 | 1316 | 1324 | - | TATCCCACC |
