# Supplementary material for: Exosome-transmitted long noncoding RNA SNHG1 promotes prostate cancer bone metastasis via YBX1/MMP16 axis
Source: Cell Death Discov. 2026 Jan 8;12:7. doi: 10.1038/s41420-025-02855-5 (PMC12783806; doi:10.1038/s41420-025-02855-5)
Supplement: Supplementary file 12 — Supplemental Methods [file 41420_2025_2855_MOESM12_ESM.docx]

1. **Sequences of primers used for RT-qPCR in this study**

| lncRNA SNHG1 | Forward (5’-3’) | | CTGTTCCCGTCATGAGCCTT | | |
| --- | --- | --- | --- | --- | --- |
|  | Reverse (5’-3’) | | GCAAGGCCCTGAATGAGCTA | | |
| YBX1 | Forward (5’-3’) | | GGGGACAAGAAGGTCATCGC | | |
|  | Reverse (5’-3’) | | CGAAGGTACTTCCTGGGGTTA | | |
| MMP16 | Forward (5’-3’) | | AGCACTGGAAGACGGTTGG | | |
|  | Reverse (5’-3’) | | CTCCGTTCCGCAGACTGTA | | |
| ENPP2 | | Forward (5’-3’) | | ACTTTTGCCGTTGGAGTCAAT |  |
|  |  | Reverse (5’-3’) | | GGAGTCTGATAGCACTGTAGGA |  |
| β-actin | Forward (5’-3’) | | CATGTACGTTGCTATCCAGGC | | |
|  | Reverse (5’-3’) | | CTCCTTAATGTCACGCACGAT | | |
| GAPDH | Forward (5’-3’) | | GGAGCGAGATCCCTCCAAAAT | | |
|  | Reverse (5’-3’) | | GGCTGTTGTCATACTTCTCATGG | | |
| U6 | Forward (5’-3’) | | CTCGCTTCGGCAGCACA | | |
|  | Reverse (5’-3’) | | AACGCTTCACGAATTTGCGT | | |

1. **Sequences of primers used for ChIP-qPCR in this study**

| MMP16 promoter 1 | Forward (5’-3’) | TGGGTCTTGGGTCAAGTTGG |
| --- | --- | --- |
|  | Reverse (5’-3’) | TGGGTGAGTTGGAAAGGCAA |
| MMP16 promoter 2 | Forward (5’-3’) | AGCTGCAAAGTCCCATTCCT |
|  | Reverse (5’-3’) | GGAAAGAAAAGCAGGCGAGG |

1. **Sequences of siRNA and shRNA in this study**

| SNHG1 siRNA 1 | Sense (5’-3’) | GGGUAUUUCAGAUGUACCUUATT |
| --- | --- | --- |
|  | Anti-sense (5’-3’) | UAAGGUACAUCUGAAAUACCCTT |
| SNHG1 siRNA 2 | Sense (5’-3’) | GCAGACACAGAUUAAGACACUTT |
|  | Anti-sense (5’-3’) | AGUGUCUUAAUCUGUGUCUGCTT |
| YBX1 siRNA 1 | Sense (5’-3’) | CGGUUUAGUCAUCCAACAAGATT |
|  | Anti-sense (5’-3’) | UCUUGUUGGAUGACUAAACCGTT |
| YBX1 siRNA 2 | Sense (5’-3’) | AUGUAAGGAACGGAUAUGGUUTT |
|  | Anti-sense (5’-3’) | AACCAUAUCCGUUCCUUACAUTT |
| Ctrl  siRNA | Sense (5’-3’) | UUCUCCGAACGUGUCACGUTT |
|  | Anti-sense (5’-3’) | ACGUGACACGUUCGGAGAATT |
| SNHG1 shRNA 1 | (5’-3’) | CACCGGGTATTTCAGATGTACCTTACGAATAAGGTACATCTGAAATACCCTTTT |
| SNHG1 shRNA 2 | (5’-3’) | CACCGCAGACACAGATTAAGACACTCGAAAGTGTCTTAATCTGTGTCTGCTTTT |

1. **Antibodies used in this study**

| **Gene/target** | **Company** | **Catalog Number** |
| --- | --- | --- |
| CD9 | CST | 13174 |
| HSP70 | CST | 4876 |
| Calnexin | CST | 2433 |
| COL1A1 | Proteintech | 67288-1-Ig |
| Alkaline Phosphatase (ALP) | HUABIO | ET1601-21 |
| RUNX2 | Proteintech | 20700-1-AP |
| Osteocalcin | Abcam | ab93876 |
| GAPDH | Proteintech | 60004-1-Ig |
| YBX1 | Proteintech | 20339-1-AP |
| MMP16 | Abcam | ab313410 |
| ENPP2 | Proteintech | 68724-1-Ig |
| Tubulin | Proteintech | 66031-1-Ig |
| Histone H3 | HUABIO | M1309-1 |
| HRP-anti-Rabbit-IgG | Proteintech | SA00001-2 |
| HRP-anti-mouse-IgG | Proteintech | SA00001-1 |
| Alexa Fluor® 594  anti-mouse-IgG | Abcam | ab150116 |
| Alexa Fluor® 488  anti-Rabbit-IgG | Abcam | ab150073 |

1. **Brief description of supplementary files**

**Table S1**

Using a ChIRP (Chromatin Isolation by RNA Purification) probe targeting lncRNA SNHG1, compared to a control probe, followed by mass spectrometry to identify significantly enriched proteins.

**Table S2**

Annotation and background description of significantly differentially expressed proteins in Table S1.

**Table S3**

Among the top proteins with the highest fold change in CHIRP-MS, use the lncPro ([http://bioinfo.bjmu.edu.cn/lncpro](http://bioinfo.bjmu.edu.cn/lncpro/) ) or catRAPID (<http://service.tartaglialab.com/page/catrapid_group> ) tools to evaluate and rank the interactions between the top proteins and SNHG1.

**Table S4**

Using the cBio Cancer Genomics Portal ([http://cbioportal.org](http://cbioportal.org/)), we analyzed the mRNA expression (FPKM capture) of prostate cancer in primary or metastatic tissues to identify specific expression patterns in two datasets: Metastatic Prostate Adenocarcinoma (SU2C/PCF Dream Team, PNAS 2019) and Metastatic Prostate Cancer (SU2C/PCF Dream Team, Cell 2015).

**Table S5**

Using the UCSC RefSeq database, the nearest gene annotations were performed for the CHIRP peaks enriched by the SNHG1 probe located within -2Kb to +2Kb around the gene transcription start site (TSS).

**Table S6**

Using the JASPAR database (<http://jaspar.genereg.net/>), binding sites for the transcription factor YBX1 were predicted within the ±2k region of the transcription start site (TSS) of the MMP16 and ENPP2 gene promoters, with a relative score threshold greater than 90%.
