# Supplementary material for: Exosome-transmitted long noncoding RNA SNHG1 promotes prostate cancer bone metastasis via YBX1/MMP16 axis
Source: Cell Death Discov. 2026 Jan 8;12:7. doi: 10.1038/s41420-025-02855-5 (PMC12783806; doi:10.1038/s41420-025-02855-5)
Supplement: Supplementary file 13 — Original Data of Western blot [file 41420_2025_2855_MOESM13_ESM.docx]

**Figure 2C**



CD9



HSP70


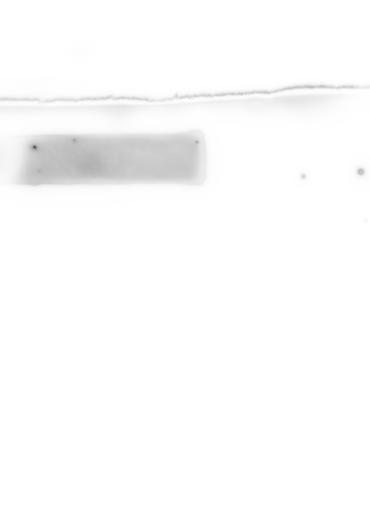
calnexin

**Figure 4F**


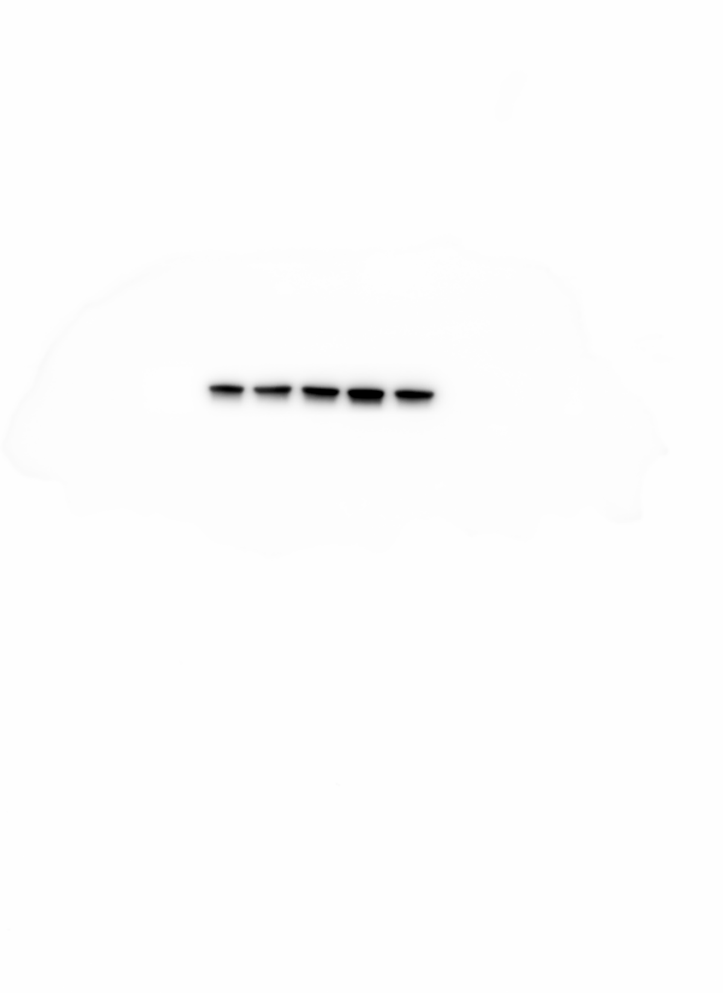
COL1A1


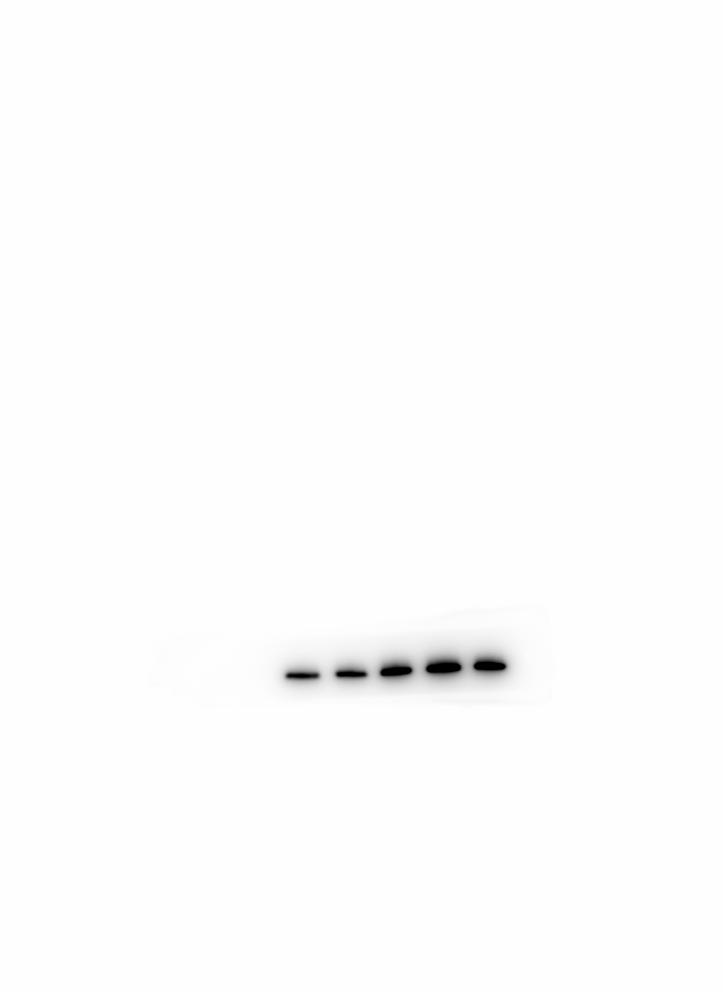
ALP


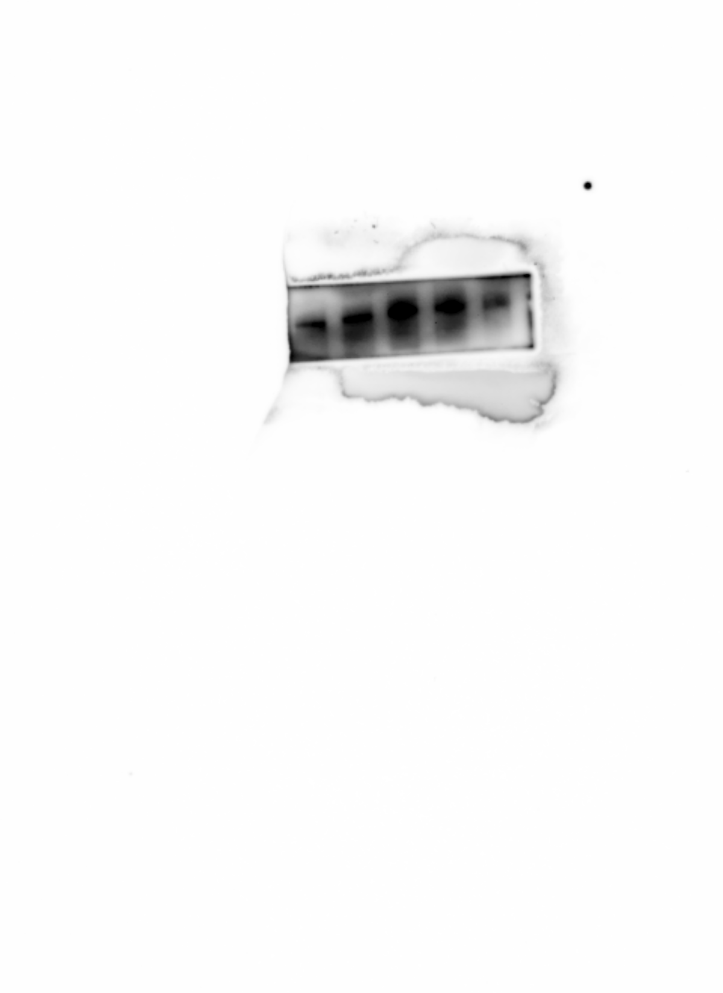
RUNX2


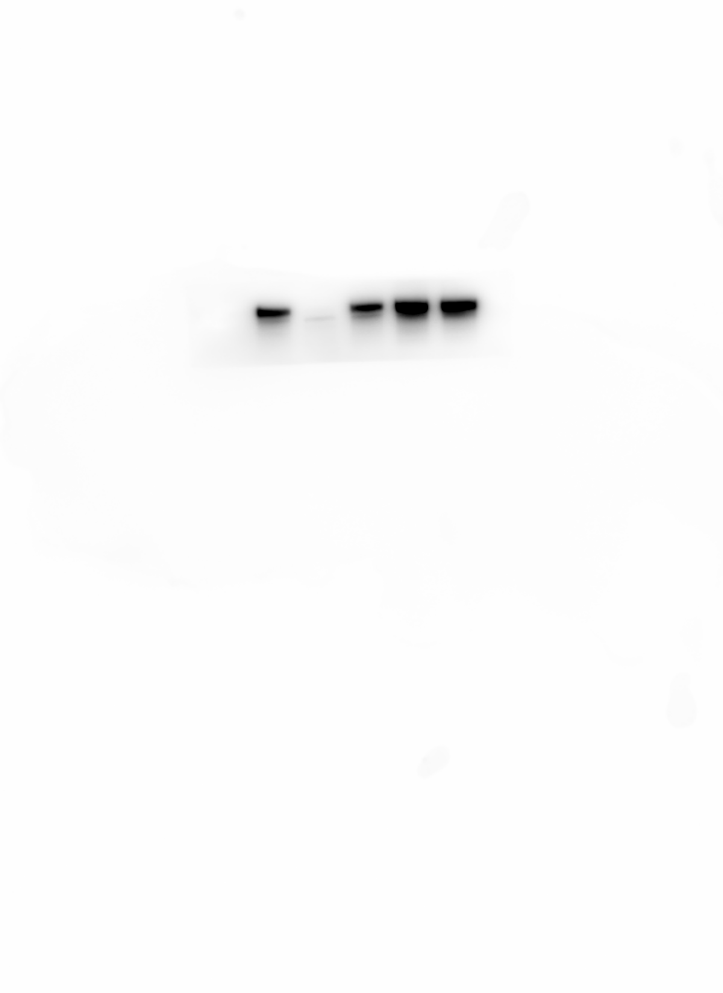
Osteocalcin


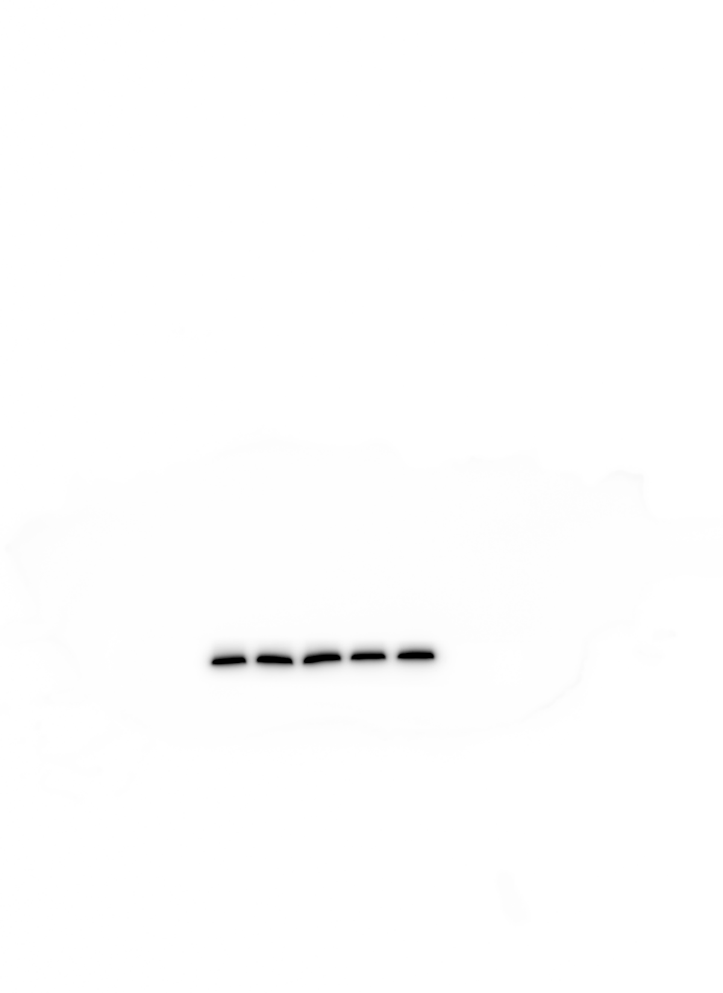
GAPDH

**Figure 5D**



YBX1


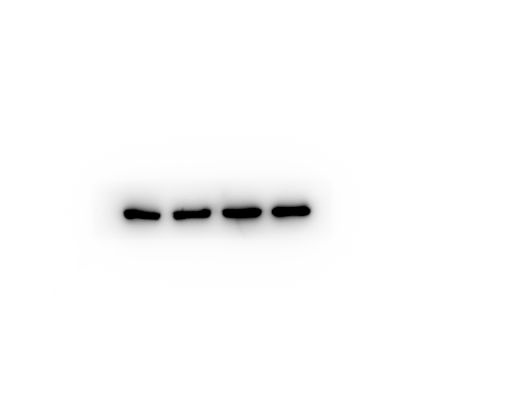
GAPDH

**Figure 5G**


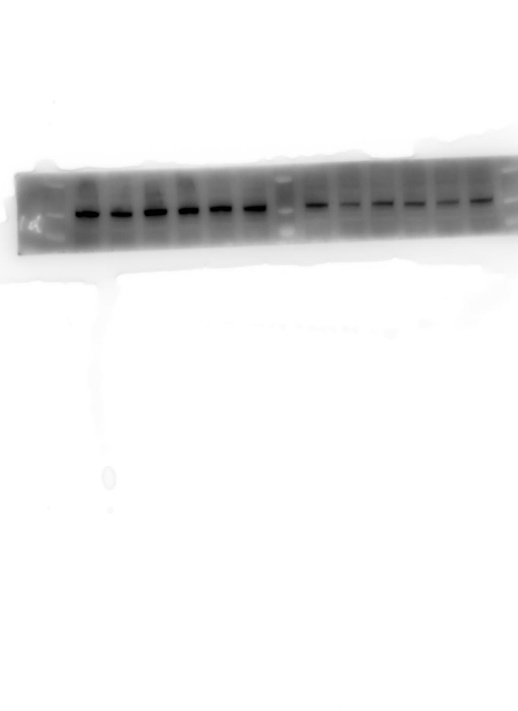
GAPDH


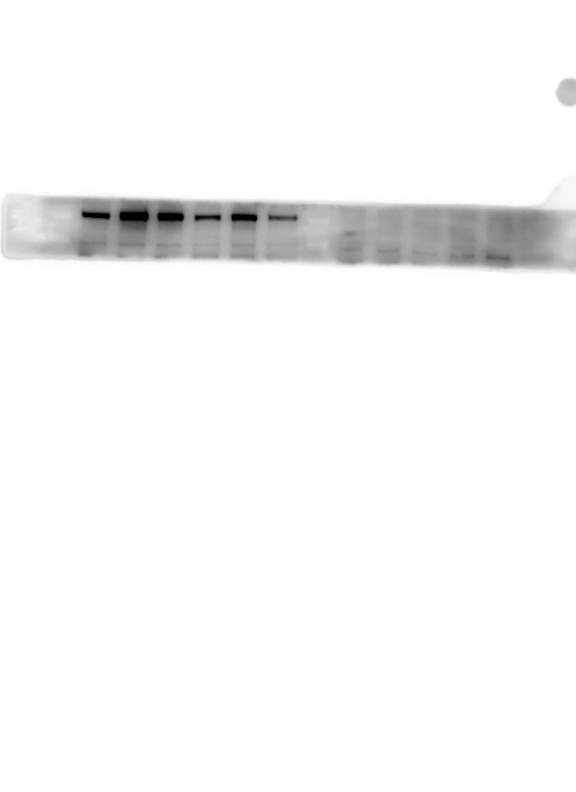
H3
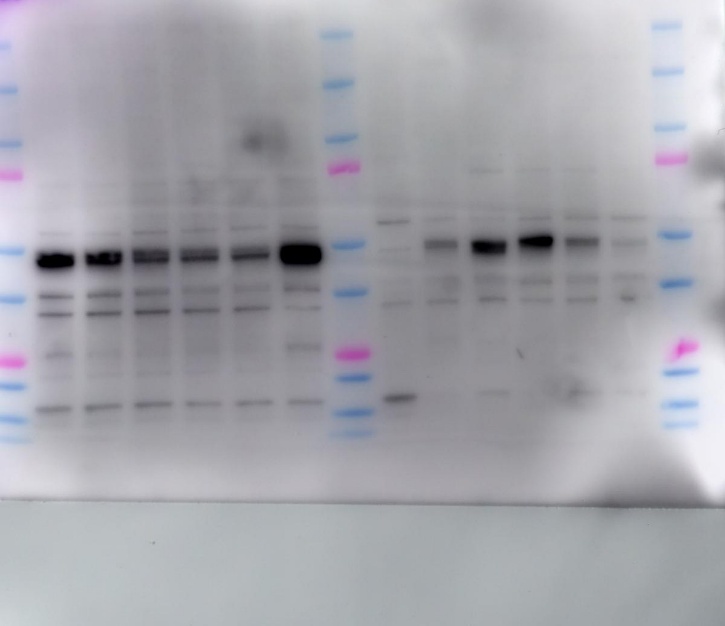
YBX1

**Figure 6C**



MMP16


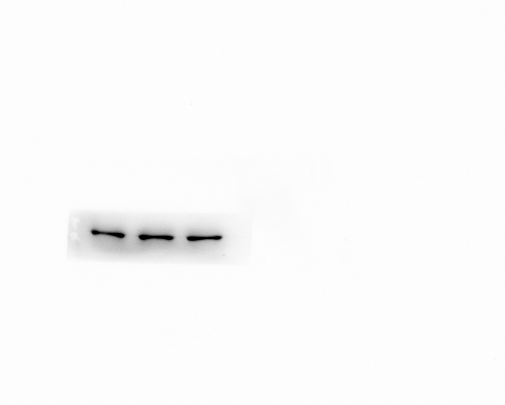
GAPDH



ENPP2


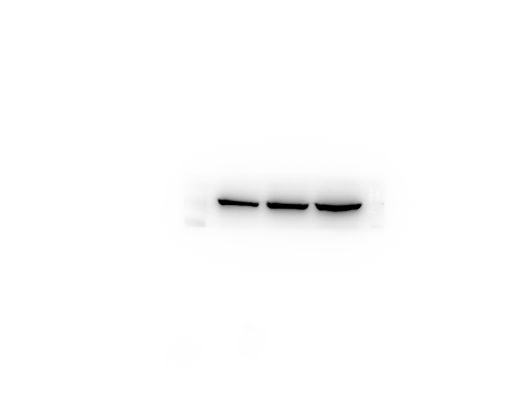
Tubulin

**Figure 6F**


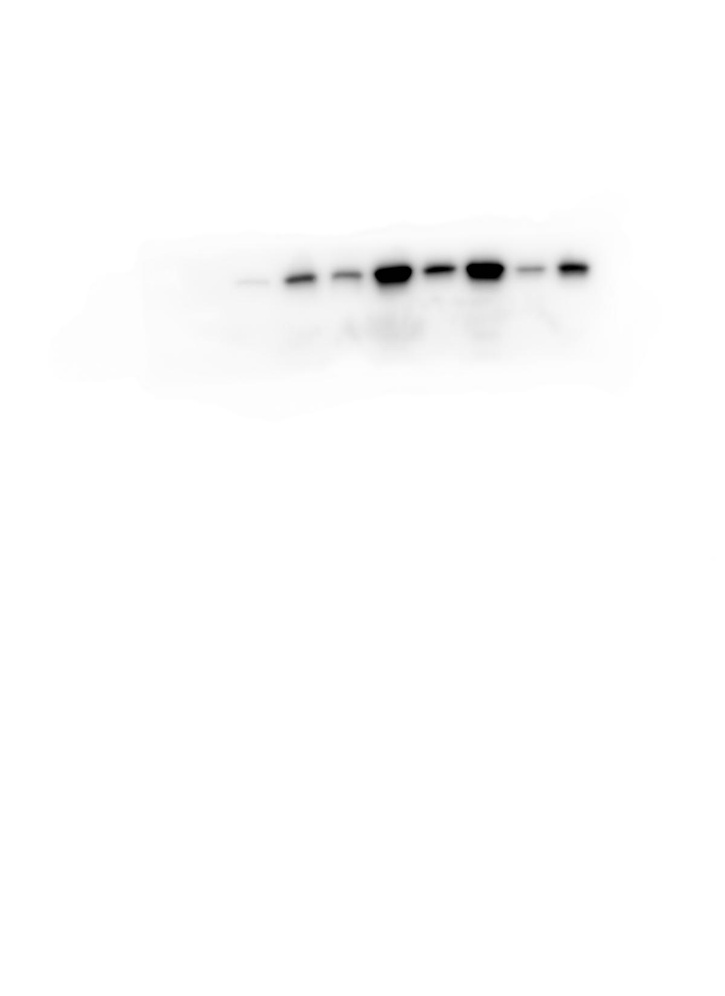
MMP16


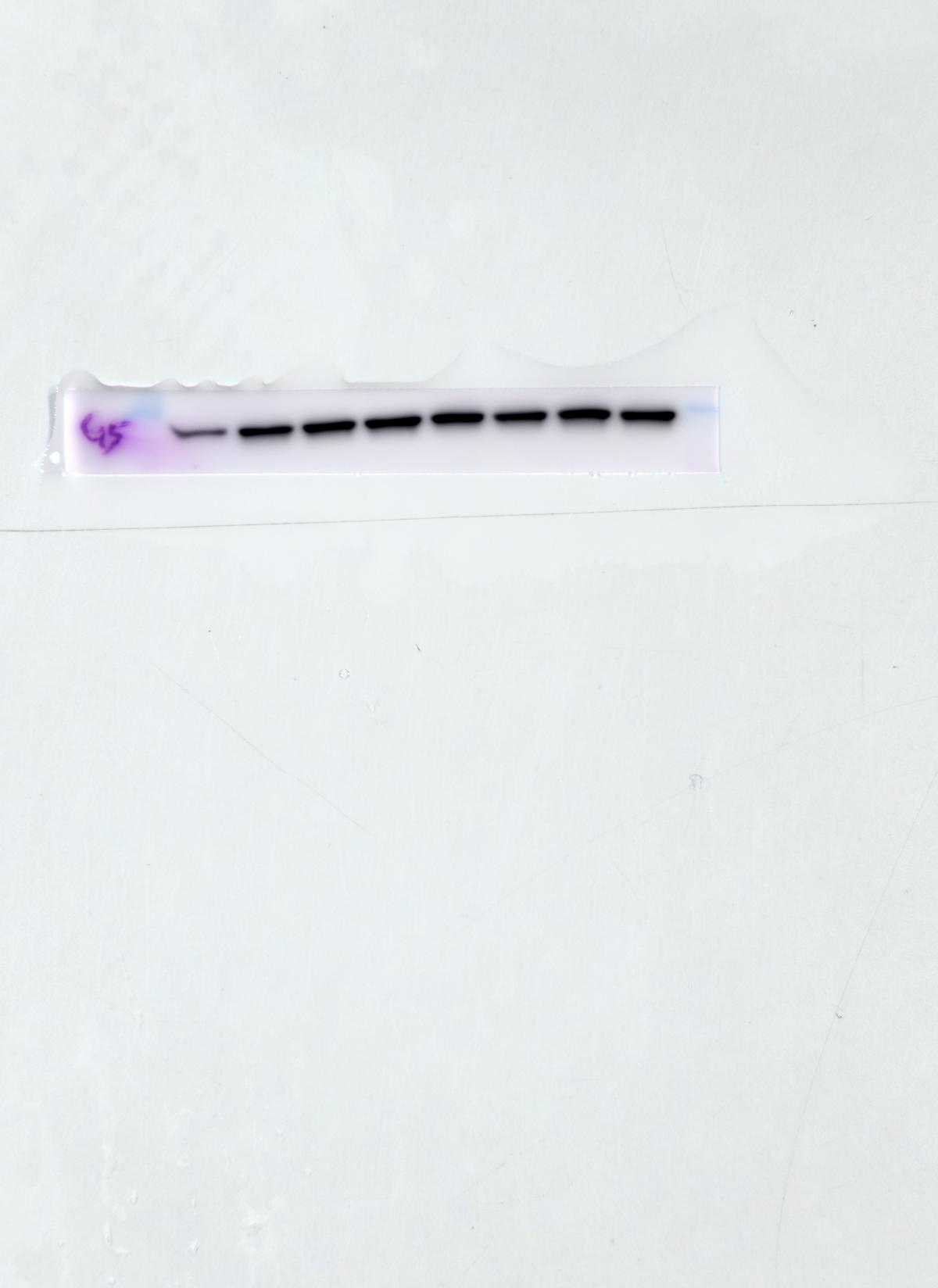
GAPDH


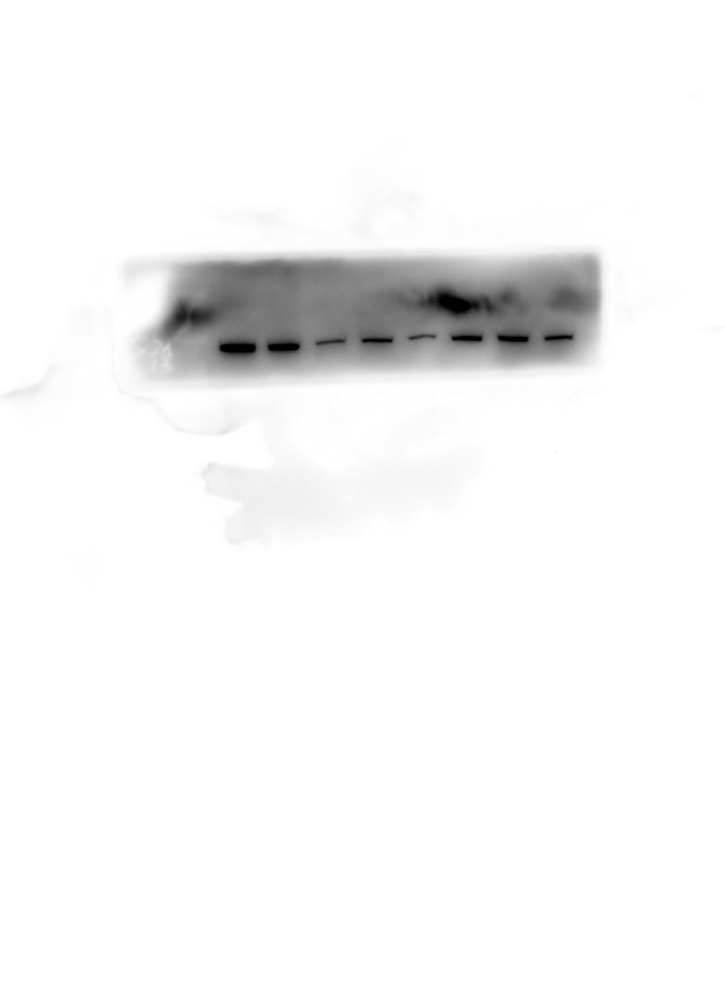
YBX1

**Figure 6K**

**
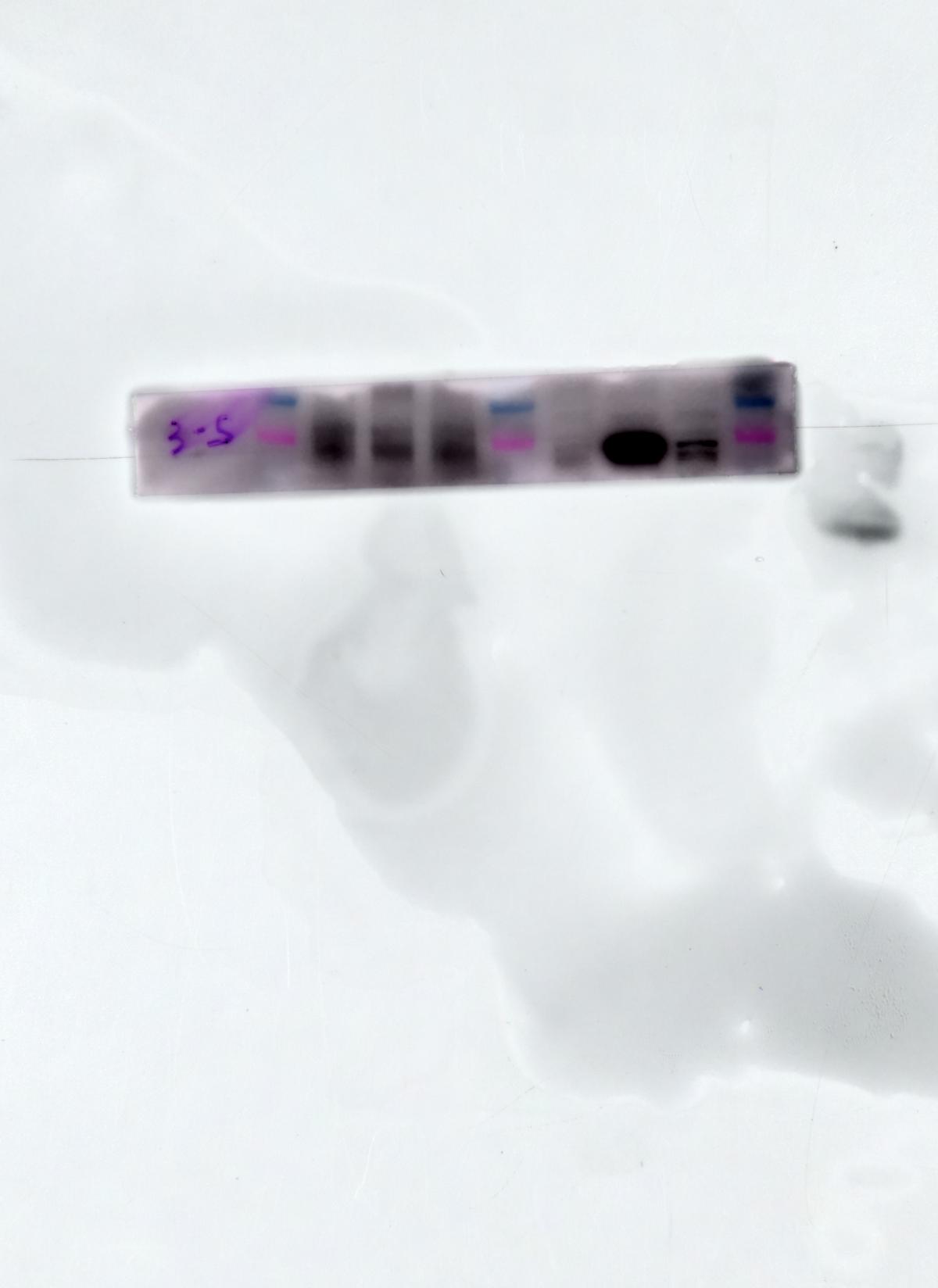
**MMP16


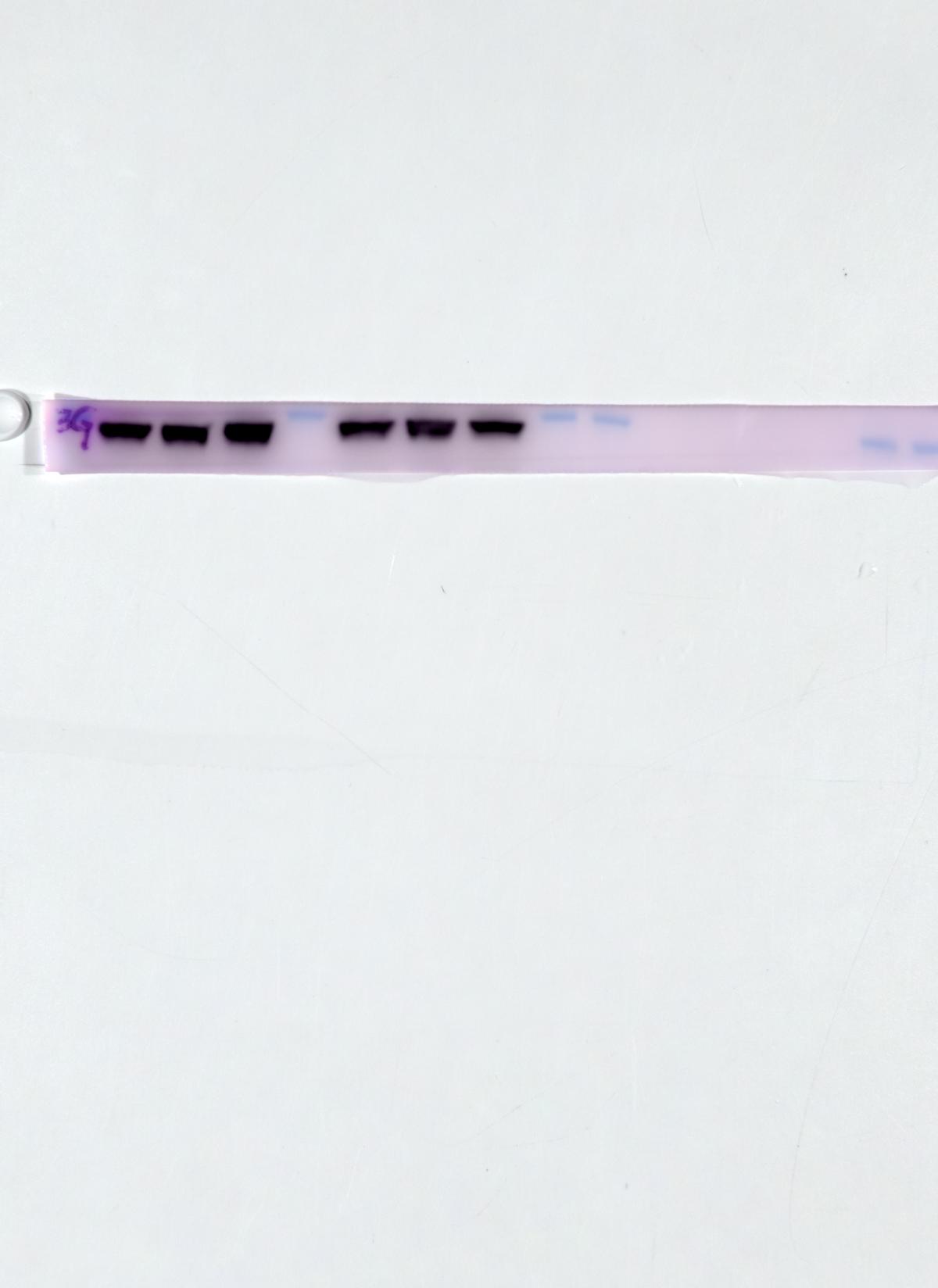
YBX1


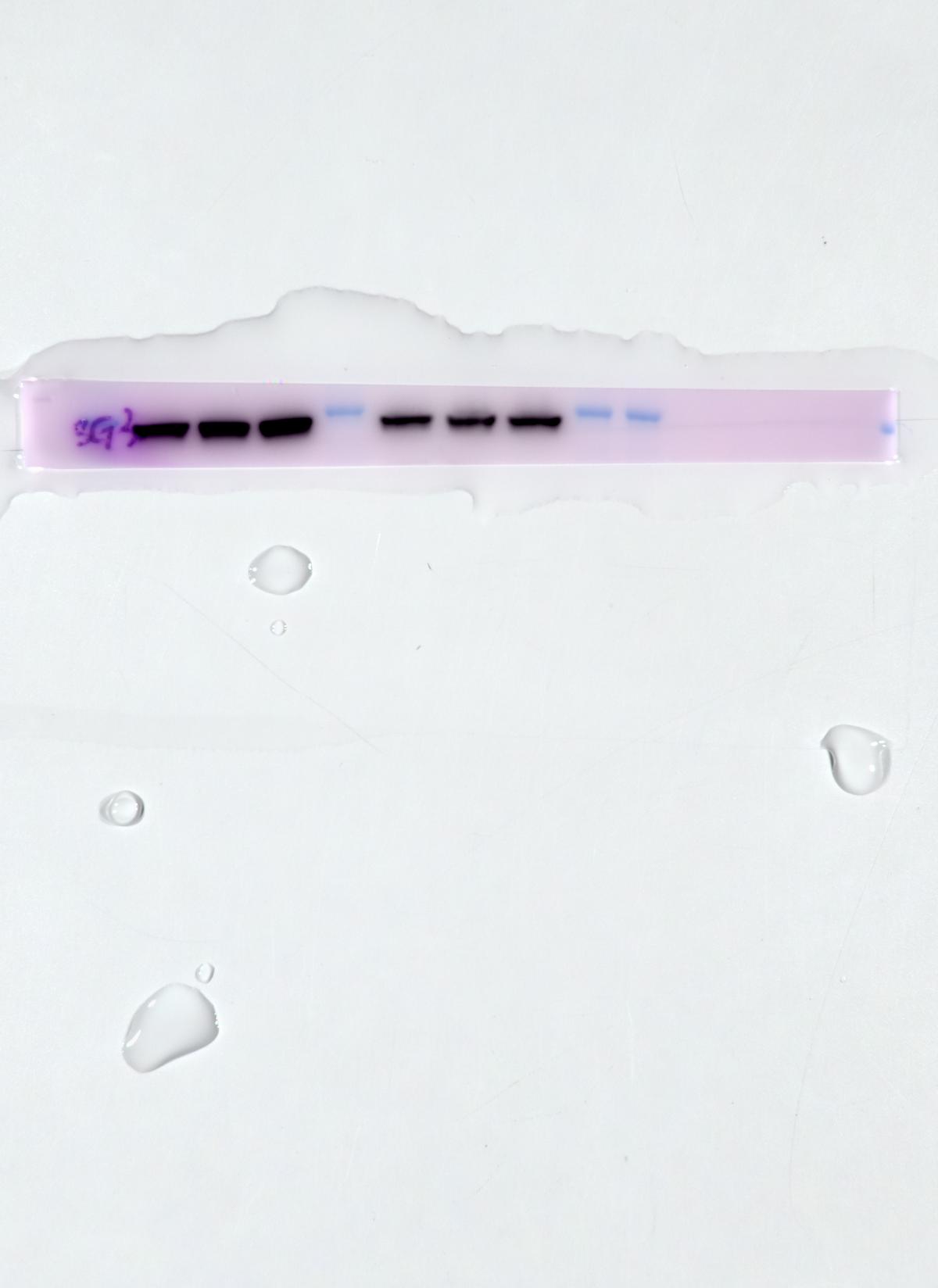
GAPDH

**Figure 7F**


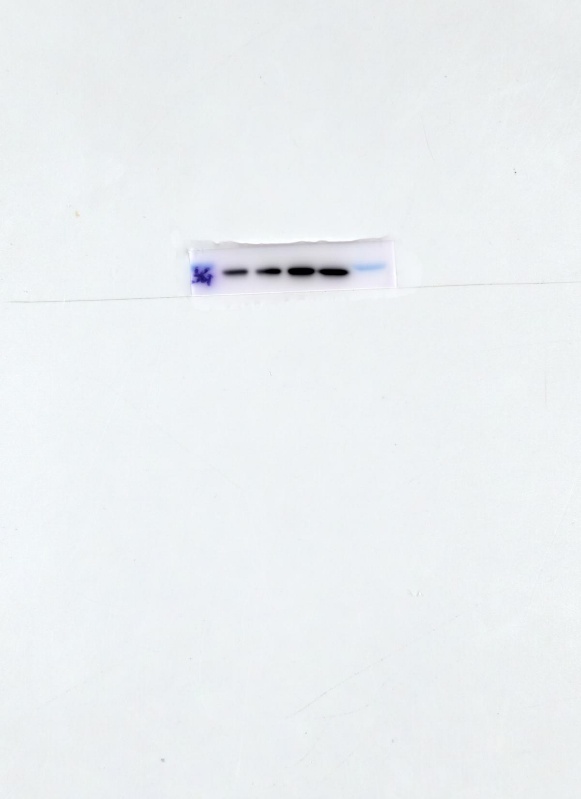
YBX1


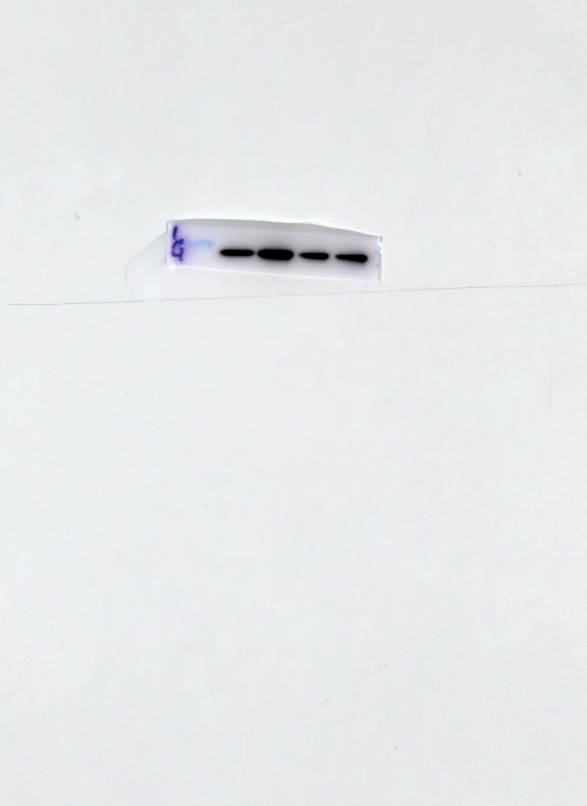
Osteocalcin


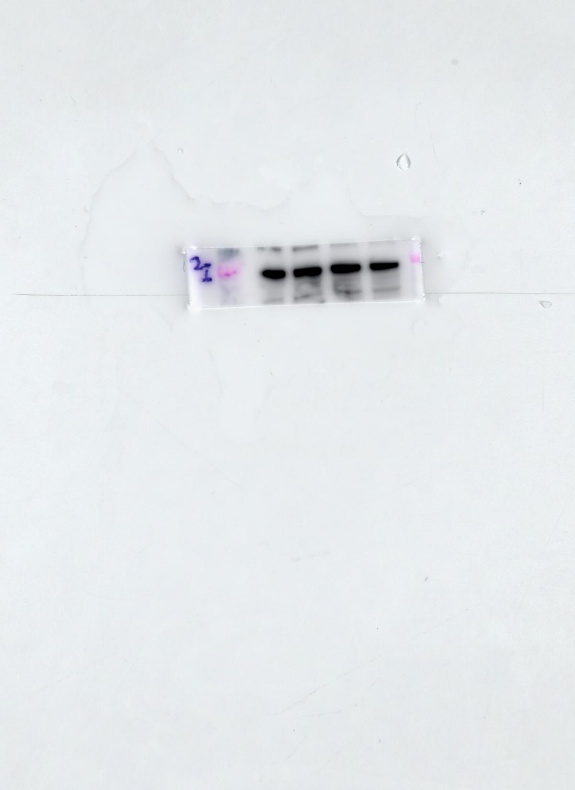
RUNX2


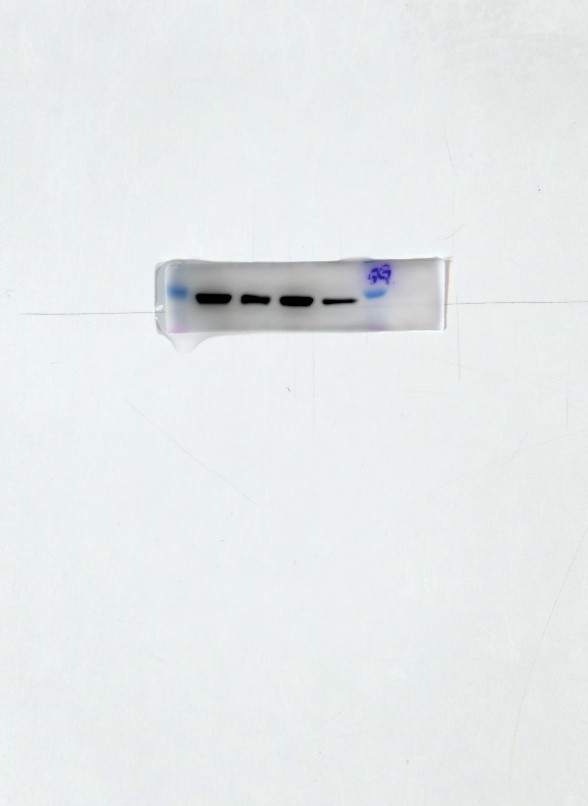
MMP16


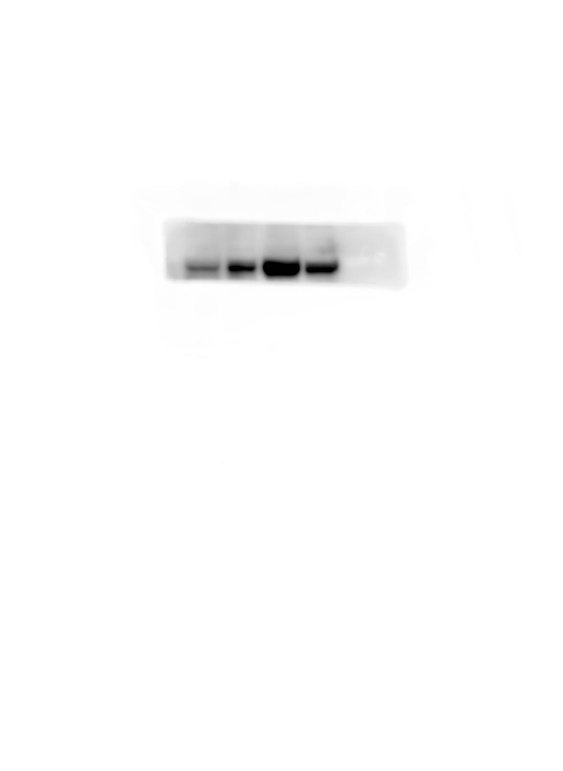
ALP


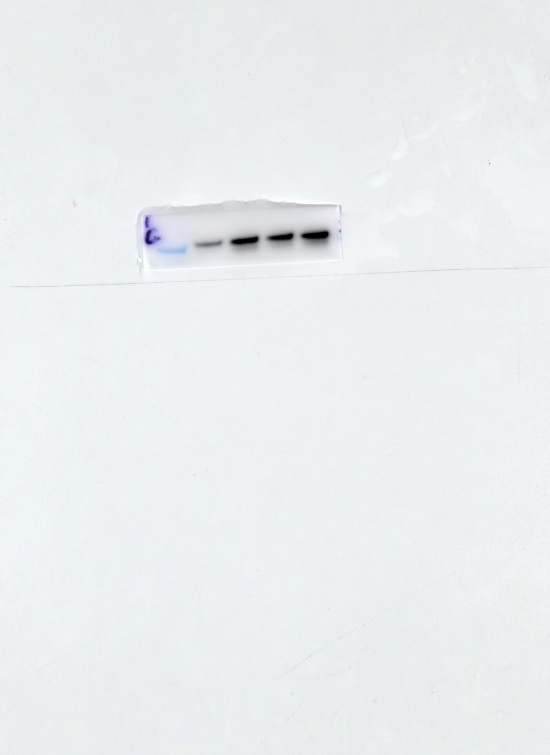
Tubulin
